# Using systems perspectives in evidence synthesis: A methodological mapping review

**DOI:** 10.1002/jrsm.1595

**Published:** 2022-08-18

**Authors:** Quan Nha Hong, Mukdarut Bangpan, Claire Stansfield, Dylan Kneale, Alison O'Mara‐Eves, Leonie van Grootel, James Thomas

**Affiliations:** ^1^ EPPI‐Centre, UCL Social Research Institute University College London London UK; ^2^ Rathenau Instituut The Hague The Netherlands

**Keywords:** complex adaptive systems, complex systems, evidence synthesis, systematic reviews, systems perspective, systems thinking

## Abstract

Reviewing complex interventions is challenging because they include many elements that can interact dynamically in a nonlinear manner. A systems perspective offers a way of thinking to help understand complex issues, but its application in evidence synthesis is not established. The aim of this project was to understand how and why systems perspectives have been applied in evidence synthesis. A methodological mapping review was conducted to identify papers using a systems perspective in evidence synthesis. A search was conducted in seven bibliographic databases and three search engines. A total of 101 papers (representing 98 reviews) met the eligibility criteria. Two categories of reviews were identified: (1) reviews using a “systems lens” to frame the topic, generate hypotheses, select studies, and guide the analysis and interpretation of findings (*n* = 76) and (2) reviews using systems methods to develop a systems model (*n* = 22). Several methods (e.g., systems dynamic modeling, soft systems approach) were identified, and they were used to identify, rank and select elements, analyze interactions, develop models, and forecast needs. The main reasons for using a systems perspective were to address complexity, view the problem as a whole, and understand the interrelationships between the elements. Several challenges for capturing the true nature and complexity of a problem were raised when performing these methods. This review is a useful starting point when designing evidence synthesis of complex interventions. It identifies different opportunities for applying a systems perspective in evidence synthesis, and highlights both commonplace and less familiar methods.

**Highlights**

**What Is Already Known**

- Systems perspectives provide a holistic way of thinking and allow the study of complex systems, that is, systems with many interdependent elements interacting dynamically and in a nonlinear manner.
- Researchers have been advocating applying systems perspectives in evidence synthesis to better understand intervention complexity.

**What Is New**

- Two categories of papers using a systems perspective were identified: (1) reviews using a “systems lens” to frame the topic, generate hypotheses, select studies, guide the analysis and/or interpretation of findings, and (2) reviews using systems methods to identify, rank and select elements, analyze interactions, develop models, and forecast needs.
- There were various forms of implementation within each of the two categories.
- Some of the methods may be unfamiliar to evidence synthesists, including systems dynamics, agent‐based modeling, and soft systems methodology.

**Potential Impact for RSM Readers Outside the Authors' Field**

- This review is a useful starting point to help reviewers plan a review of complex interventions using a systems perspective. It identifies different opportunities for applying systems perspectives in evidence synthesis and highlights commonplace and less familiar methods for implementation.

## 1 INTRODUCTION

Reviewing complex interventions is challenging because they often include a large number of elements and complex pathways.^1^ The 2019 edition of the Cochrane Handbook for Systematic Reviews of Interventions suggests that three perspectives on intervention complexity are possibly applicable in systematic reviews: (a) a focus on the components of an intervention; (b) a focus on interactions between components of an intervention and/or between an intervention and other aspects (e.g., participants, context); and (c) a focus on the system within which the intervention is introduced (i.e., impact of an intervention on the dynamics of a system or of the system on an intervention).^2^ Several methods to address the first two perspectives have been suggested in the literature and are currently used, such as network meta‐analysis^3^ and causal chain analysis.^4^ However, although researchers have been advocating using a systems perspective to review interventions complexity for some time,^2^, ^5^, ^6^, ^7^, ^8^ only a few papers on systems methods in evidence synthesis have been published.

A *system* is defined as “an interconnected set of elements that is coherently organized in a way that achieves something.”^9^ This definition highlights three important components of a system: (a) its elements (i.e., entities that characterize and make up a system), (b) its interconnections (i.e., the way elements feed back into, and are related to, each other) and (c) its purposes (i.e., what a system does). A system cannot be determined or explained by its elements alone since it is viewed as a whole that is more than the sum of its parts.^9^ A complex system has been defined as a system that has a large number of interdependent elements and includes several properties such as nonlinear causation, emergent outcomes, feedback loops, and coevolution.^10^, ^11^, ^12^, ^13^ Complex systems can involve several actors from different organizations, sectors, and levels (e.g., micro, meso, macro). The complex adaptive system, a special case of complex system, consists of a system that can evolve and adjust over time.^14^ Table 1 defines some characteristics that have been attributed to complex systems.

**TABLE 1:** Attributes of complex systems

Attributes	Definition
Adaptation	The system adjusts and readjusts to changes in the environment over time. Similar concepts: dynamic, evolution, and constantly changing.
Coevolution	A system influences and is influenced by other systems. Similar concepts: dependencies and tightly linked.
Counter‐intuitive	Cause and effect are often distant in time and space. Effective solutions can work in some settings but not in others.
Emergence	Spontaneous, unplanned, or unpredictable behaviors arise from the interaction within or between elements of a system.
Feedback loops	Situations in which elements reinforce or balance other elements and influence subsequent actions.
System history/memory	The history of a system influences its nature and affects its evolution.
Multiple interacting elements	Complex systems are characterized by a large number of elements that are interconnected. These elements can be known or unknown.
Multiple agents	Different actors are involved within the system to contribute to solution making.
Non‐linearity	Not all relationships within a system can be arranged along an input–output proportional line.
Resistant to change	Failure to change may occur because the complexity of the system overwhelms one's ability to understand it and seemingly obvious solutions may fail or worsen a situation.
Self‐organization	The dynamics of a system arise spontaneously from its internal structure.

*Source*: Definitions adapted from: Petticrew et al.,^6^ Brainard and Hunter,^10^ Rutter et al.,^13^ Plsek and Greenhalgh,^16^ Sturmberg et al.^30^ Wilkinson et al.^31^ De Savigny and Adam,^80^ Penney et al.^82^ Richardson et al.^83^

Systems *perspectives* involve a holistic way of seeing the world.^15^ Systems researchers are interested in understanding how the different elements of a system interact with each other and how these influence the system's behaviors.^9^, ^16^ In a systems perspective, interventions are seen as nested within a wider system that cannot be fully understood by only using linear causal models and by examining their components in isolation.^4^, ^13^, ^17^ A systems perspective focuses on the context in which an intervention is introduced, the relationships and interactions between the actors and its context, and the changes within the systems itself.^6^ Thus, when analyzing a complex system, it is necessary to analyze the dynamics of the system in order to forecast its possible evolution in the future.^18^ For example, the COVID‐19 pandemic can be seen as a major perturbation in our complex biological and social systems with multiple and multifaceted elements that interact with one another in nonlinear and unpredictable ways.^10^, ^13^ The decisions made to control the pandemic do not follow a linear process, but are the result of feedback loops, such as policy responses to evidence on the spread of the virus. For example, the success of policies that recommend the wearing of face coverings, are not only the result of deterministic processes—such as the degree to which the covering prevents droplets traveling away from an individual. They are also influenced by shifting social and political norms that, in this case, mediate and moderate whether face coverings are worn at all. Moreover, there are complex social interactions between one policy—face coverings—and people's responses to other social policy, such as social distancing regulations. A systems perspective aims to permit the investigation of these types of multiple and interacting elements.

Systems perspectives have evolved since the 1950s.^19^ A historical overview of different systems approaches has identified three main waves: hard, soft, and critical systems approaches^15^, ^19^, ^20^ (see Table 2 for a summary of their main characteristics). The first wave contains *hard systems approaches that were mainly* developed during the 1950s and 1960s in the fields of operational research and systems engineering. The aim of these approaches is to “understand how things are connected, by what, to what and with what consequence.”^20^ Hard systems approaches often use computer simulations to build models to represent the interaction between components of a system. During the 1970s, a second wave emerged with the development of *soft systems* approaches to explore the implication of different perspectives within a system on the interrelationships between elements.^15^ They “put emphasis on dialogue, conflict resolution, mutual appreciation, and inter‐subjective construction of meaning.”^19^ The purpose of doing this is to enable communication between different groups of individuals in order to identify potential solutions to improve the situation.^15^ By the 1980s, a third wave is seen with the development of *critical systems* approaches to address the need to be critical about defining system boundaries and to establish boundaries within which critique can be conducted.^15^ Three core values are essential in critical systems approaches: critique (e.g., being critical about the chosen methods and theories), emancipation (e.g., freeing the systems and its individuals from oppression and coercion), and pluralism (e.g., using a combination of methodologies).^21^ Also, critical systems approaches are interested in addressing coercive contexts, especially contexts of marginalization, conflicts, ethical issues, and power relations.^21^, ^22^

**TABLE 2:** Main types of systems approaches

Approach	Ontology	Epistemology	Teleology (purpose)	Examples of methods
Hard	Realism	Postpositivism	Control Enables making sense of relationships between elements	Agent‐based modeling Network analysis Systems dynamics
Soft	Relativism	Constructivism/Interpretivism	Appreciation Enables communication and engaging contrasting perspective	Inquiring system design Strategic assumption surface testing Soft systems methodology
Critical	Relativism	Constructivism/Critical idealism	Emancipation Enables reconciliation of ethical issues and power relations	Community operational research System of systems methodologies Systemic intervention

*Source*: Adapted from Reynolds.^15^

Systems perspectives have mainly been applied in primary research. For example, various systematic reviews of primary research that used systems perspectives can be found in the literature for a wide diversity of fields such as public health,^23^ education,^24^ and management.^25^ More recently, researchers have been advocating applying systems perspectives in evidence synthesis to better understand intervention complexity.^6^ However, it is still not clear how systems perspectives might be applied in evidence synthesis and to what extent systems perspectives provide different analytical possibilities compared with existing methods addressing “complexity.”^1^ Thus, the aim of this project was to understand how and why systems perspectives have been applied in evidence synthesis.

## 2 METHODS

A methodological mapping review was performed. A methodological review synthesizes information on the conduct, analysis, and reporting of research methods.^26^ A “mapping” of the literature consists of collating, describing, and cataloging available evidence on a topic.^27^ A descriptive map provides information of the available studies across different topic areas.^28^ In this review, this allowed us to identify and describe the system approaches used in evidence synthesis.

### 2.1 Search strategy

Inspired by existing search strategies used in systematic reviews on systems perspectives of primary research,^10^, ^23^, ^29^, ^30^, ^31^ one author drafted a search strategy in consultation with an information specialist. It was then adapted for seven databases that collectively contain research in healthcare, social sciences, and business: Medline (OVID), Embase (OVID), PsycInfo (OVID), CINAHL (EBSCO), Social Sciences Citation Index (Web of Science), Public Health Database (ProQuest), and ABI/INFORM (ProQuest). Key terms on systems perspectives were combined with terms for evidence synthesis (see Table 3, e.g., the search strategy in Medline). The search was carried out from inception of each database to May 2019. This was supplemented by focused searching of three online search engines during November 2019: Google Scholar, Microsoft Academic, and BASE (Bielefeld Academic Search Engine). The aim of these later searches was to boost the identification of evidence synthesis outside health that could have been missed as the earlier databases predominantly covered health research. Search terms for the supplementary searches included: “systematic reviews” and “complexity theory”; “systematic reviews” and “systems thinking,” excluding health. All potentially relevant records were transferred to EPPI‐Reviewer^32^ for eligibility assessment, data extraction, and synthesis. During the data extraction of included papers, a search in Google Scholar was performed to identify companion papers of included studies, that is, papers on the same study published elsewhere (such as Part 1 and Part 2 papers, or dissertations).

**TABLE 3:** Search strategy (Medline)

Concepts	Search terms
Systems perspective	1. (“complexity science?” or “complexity theor*” or “system? approach*” or “System? change*” or “System? model*” or “System? perspective?” or “System? science?” or “System? thinking” or “System* theor*” or “Nonlinear* system?” or “Non linear* system?” or “Multi agent system?” or “Multiagent system?” or “agent based model*” or (Complex* adj2 system?) or (System? adj dynamic*) or (chaos adj1 theory)).ti,ab,kw.
	2. Nonlinear dynamics/
	3. Systems theory/
	4. Systems analysis/
	5. 1 or 2 or 3 or 4
Evidence synthesis	6. ((“review” and [integrat* or critical* or “mapping” or “comprehensive” or “evidence” or “research” or “literature”]) or ((“synthesis” or “systematic”) and (“evidence” or “research” or “review”))).ti.
	7. ((Systematic* adj2 review*) or “Meta analy*” or “Metaanaly*” or “metaethnograph*” or “meta‐epidemiological” or “meta‐regression” or “metasummary” or “data synthesis” or “evidence synthesis” or “metasynthesis” or “meta‐synthesis” or “narrative synthesis” or “qualitative synthesis” or “quantitative synthesis” or “realist synthesis” or “research synthesis” or “synthesis of evidence” or “thematic synthesis” or “bibliographic search” or “database search” or “electronic search” or “handsearch*” or “hand search*” or “keyword search” or “literature search” or “search term*” or “data extraction” or “narrative review” or “iterative review” or “realist review” or “scoping stud*” or “scoping review” or “systematic map*” or (mixed adj2 review*) or (mixed adj2 synthesis)).ti,ab,kw.
	8. “Systematic review”/
	9. Systematic reviews as topic/
	10. Meta‐analysis/
	11. Meta‐analysis as topic/
	12. 6 or 7 or 8 or 9 or 10 or 11
	13. 5 and 12

### 2.2 Eligibility assessment

Papers were included if they conducted an evidence synthesis in which the authors stated using a systems perspective in their review such as using a systems theory, systems method (e.g., as presented in Table 2), and/or developing a systems model. Several terms could be used to designate systems perspective such as systems sciences, systems thinking, systems theory, systems approach, complex adaptive system, complex sociotechnical system, and social‐ecological systems research. In this project, a broad definition of evidence synthesis was used: all papers that mentioned performing a literature review and described the methods used (e.g., search strategy, sources used, data extraction, and/or synthesis method) were considered for inclusion.

Papers were excluded if they were solely primary studies, nonempirical (e.g., opinion paper, editorial, commentary, summary), conference abstracts, protocols, and not written in English or in French. Moreover, conceptual or methodological papers and economic evaluations were excluded. Finally, reviews that aimed at understanding how systems perspectives were used in a specific field were excluded (e.g., review describing how systems science was applied in public health,^23^ review on health interventions that were informed by complexity science,^10^ or review on systems dynamics modeling in entrepreneurship research^33^). These papers were considered reviews on systems perspective.

A two‐stage eligibility assessment process was followed starting with the screening of the titles and abstracts of records, and then examining the full‐text papers. One reviewer was involved in the whole assessment process. A second reviewer independently screened 5% of the titles/abstracts and discussion between the reviewers was held to clarify the eligibility criteria.

### 2.3 Data extraction and synthesis

The data extracted were: year, country of the lead author, topic, aim, number of studies included, reasons for using systems perspectives, methods used, software, systems visualization, data used, and limits/challenges encountered when using systems perspective as reported by the original authors. Two reviewers independently extracted data of two papers to pretest the data extraction form. Afterward, one reviewer performed data extraction for the remaining papers.

The literature was mapped and a thematic summary was produced because the aim of the methodological mapping review was to summarize what was reported in the included papers and not to reinterpret the published data.^34^ To produce the map, data on the reviews' characteristics (e.g., year, country, and topic), and systems approaches (e.g., systems methods, software, and systems visualization) were tabulated. For the thematic summary, data on the reasons for using systems perspectives and limits/challenges encountered when using systems methods were grouped into general categories by one reviewer and checked by a second reviewer.

## 3 RESULTS

**FIGURE 1:**
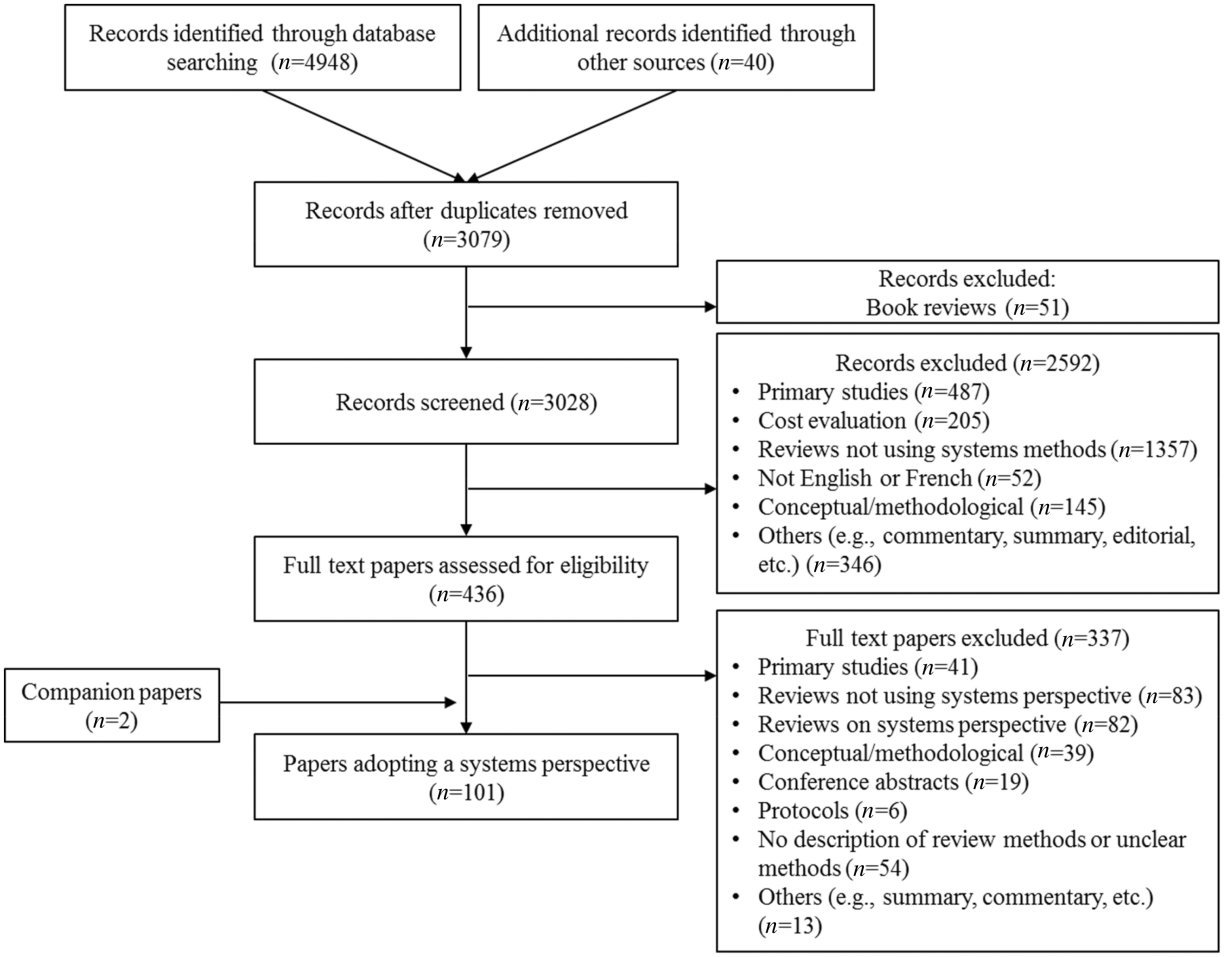
Flow diagram

A total of 3028 records were screened, 436 full‐text papers were assessed for eligibility, and 101 papers using systems perspectives were retained, representing 98 reviews (see Figure 1). The included reviews were published between 1986 and 2019, with most of them published after 2010 (*n* = 84). The first authors of the papers were affiliated with institutions from 20 different countries; United States (*n* = 38), Canada (*n* = 9), United Kingdom (*n* = 9), Germany (*n* = 7), Australia (*n* = 7), Brazil (*n* = 4), the Netherlands (*n* = 4), and Sweden (*n* = 3) were the most frequent countries. Most of the included papers addressed a topic in health (*n* = 49) followed by management and organizational studies (*n* = 15), child, adolescents, and family studies (*n* = 12), agriculture and environmental studies (*n* = 8), education (*n* = 6), information sciences (*n* = 4), physical activity (*n* = 2), urban and rural studies (*n* = 2). The number of included papers in the reviews ranged from 5 to 1012. Five were dissertations, of which two also published their literature review in a scholarly journal. Around a fifth of the included papers also used other data such as surveys, interviews, consultations with experts, and case studies (*n* = 20).

For this methodological mapping review, two general categories of papers using a systems perspective were identified. The first category comprises reviews using a systems lens and/or developing a systems model (*n* = 76) (see File S1). Several papers in this category mentioned using a systems theory or framework to guide the review. The cited theories and frameworks were: (socio‐) ecological systems theory developed by Bronfenbrenner (*n* = 28), complex adaptive systems (*n* = 9), complexity theory (*n* = 8), systems thinking (*n* = 6), systems theory or approach (*n* = 5), general systems theory by von Bertalanffy (*n* = 3), Rasmussen's Risk Management Framework (*n* = 3), family systems theory (*n* = 3), complex dynamic systems theory (*n* = 2), open system theory (*n* = 2), socio‐technical systems (*n* = 2), chaordic systems thinking (*n* = 1), health systems theory (*n* = 1), developmental system theory (*n* = 1), and Cynefin framework (*n* = 1).

### 3.1 System lens

A systems theory was used to frame a topic, define the concepts, identify variables, develop hypotheses, inform the selection of studies, code data, guide the analysis, and interpretation of results. The majority of these reviews used a systems framework to categorize or map the literature, to perform narrative synthesis, and to organize the findings. For example, Hong et al.^35^ used the ecological systems theory to examine how the individual characteristics, micro‐, meso−/exo‐, macro‐, and chrono‐systems level factors can influence or inhibit substance use among Asian American youth. Other synthesis methods were also mentioned such as content analysis (*n* = 4),^36^, ^37^, ^38^, ^39^ realist synthesis (*n* = 3),^40^, ^41^, ^42^ framework synthesis (*n* = 2),^43^, ^44^ meta‐synthesis (*n* = 2),^45^, ^46^ thematic synthesis (*n* = 2),^47^, ^48^ and meta‐analysis (*n* = 1).^49^ For example, McLain et al.^41^ conducted a realist synthesis on fisheries' property regimes in which the socioecological framework was used as a theory for change framework to inform the context‐mechanism‐outcome (C‐M‐O) analysis. Also, one review used meta‐analysis to examine a systems model that represents the causal factors of occupational fatigue and their interrelationships.^49^ They performed meta‐analysis to explain the direction and magnitude of relationships among the factors.

In summary, the first category of studies applied a systems lens by using a systems theory or framework to organize or frame the review. They used traditional systematic review synthesis methods such as content analysis and statistical meta‐analysis, although they were guided by the systems theory in terms of data included, variables analyzed, and so on.

In contrast, the second category comprises papers that mentioned using a specific systems method to develop a systems model (e.g., systems dynamic modeling, soft systems approach) (*n* = 22). The descriptive analyses below focus on this latter category as they could provide different analytical possibilities compared with conventional evidence synthesis techniques.

### 3.2 Systems methods

Twenty‐two reviews used specific methods to explore the interrelationships of the elements of a system and develop a system model (see File S2). A diversity of methods to analyze the collected data was identified in the included papers; some used more than one method. They can be grouped into three main categories: (a) hard systems approaches; (b) soft systems approach and (c) other methods. The following will provide some examples of how these categories of methods were used in the included papers. The definitions of the methods are summarized in Table 4.

**TABLE 4:** Definition of methods mentioned to study systems elements and their interrelations

Methods	Definition	Reviews
Agent‐based modeling	Modeling approach using “computer simulations to examine how elements of a system (agents) behave as a function of their interactions with each other and their environment.”^84^	72
Analytic network process	Method for multi‐criteria decision making that allows ranking alternatives based on sets of elements, considering dependencies and feedbacks among factors, and producing forecasts.^70^	61
“Cross‐over” modeling technique	Modeling technique that “forces the modeler to consider problems from the agent perspective, whilst providing insight into known and unknown variables such as the relationship between agents.”^69^	69
Network analysis	Method that examines relationships and flows among elements of a system with visualization, description, and statistical modeling of networks.^84^	73
Qualitative systems analysis	Involves two main steps: (a) identification and selection of system variables and (b) impact analysis, which consists of analyzing the mutual interactions among the variables.^85^	67, 71
Soft systems methodology	Action‐oriented methodology to tackle problematic and messy situations. Four activities are suggested constituting a learning cycle process: (a) find out about a problematic situation; (b) make some relevant purposeful activity models; (c) debate about the situation and find changes that are desirable and culturally feasible; (d) take action to improve the problematic situation. To build models, the CATWOE analysis (Customers, Actors, Transformation process, Worldview, Owners, Environmental constraints) is suggested.^86^	74, 75
Strategic flexibility analysis	A scenario analysis tool is used to assess the capability of a system “to respond to a dynamic environment through continuous changes and systemic actions” and to forecast future needs.^71^	67, 71
Systems dynamic	Modeling approach using computer simulation to build models that represent the dynamic complexity of a system by understanding the “interplay of feedback loops, stocks, and flows that all occur within the bounded endogenous system.”^84^	50, 51, 52, 53, 54, 55, 56, 57, 58, 59, 60, 61, 62, 63, 64, 65, 66, 67, 68
System of systems (SoS) framework	SoS is a complex system formed of several independent systems. Meta‐modeling is suggested to model the inter‐ and intra‐dependencies among and between each subsystem within a system of systems.^87^	76

#### 3.2.1 Hard systems approach

Nineteen reviews used a hard systems approach in which systems modeling approaches and computer simulations were used to construct models. The methods used were systems dynamics (*n* = 16), agent‐based modeling (*n* = 1), social network analysis (*n* = 1) and “cross‐over” modeling technique (*n* = 1).

##### Systems dynamics

Sixteen included reviews used systems dynamics to build models to represent the dynamic complexity of a system.^50^, ^51^, ^52^, ^53^, ^54^, ^55^, ^56^, ^57^, ^58^, ^59^, ^60^, ^61^, ^62^, ^63^, ^64^, ^65^, ^66^, ^67^, ^68^ In the included papers, the number of systems dynamics steps described to produce a systems model ranged from three to five. For most of the reviews using systems dynamics, the findings from the literature were generally useful for problem identification, hypothesis, and model formulation. In a first step, the literature was used to articulate the problem, identify the problem relevance, identify the purpose of the model, and understand the concepts to be studied.

In the second step, the literature was useful to identify the elements (often named variables, factors, components, or parameters), prioritize factors, determine the systems boundaries, and explore links between the elements. This allowed the development of an initial systems model named a causal loop diagram that maps variables and plots causal relationships between variables, and can generate dynamic hypotheses (*n* = 13).^50^, ^51^, ^52^, ^56^, ^57^, ^58^, ^59^, ^60^, ^62^, ^63^, ^64^, ^65^, ^67^ For example, a causal loop diagram on the influence of organic farming on the growth in food production is provided in Brzezina et al.^52^ The diagram provides information on the causal links between them through feedback loops. For instance, a feedback loop shows that maximization of profits (variable 1) can lead to reinvest in machinery (variable 2), which can increase yield (variable 3) and food production (variable 4), and thus increase profit (variable 1).

In the third step, the literature could be used to determine parameter values to be entered into simulation models. This allowed the depiction of a systems dynamics model, named a stock‐and‐flow diagram (*n* = 7).^52^, ^58^, ^59^, ^61^, ^63^, ^64^, ^68^ For example, Zammar et al.^68^ developed a systems diagram to depict the factors affecting the willingness to participate in clinical trials (flow) that regulate the amount of clinical trials (stock).

The last steps of systems dynamics involve testing, validating and calibrating the model, performing sensitivity analysis, and formulating policy implications. For these steps, other types of data collected from qualitative or quantitative methods were used, such as data from case studies,^59^, ^60^, ^61^, ^69^ mixed methods studies,^55^, ^67^ and real or synthetic data.^52^, ^53^, ^54^, ^63^

Among the 16 reviews, four provided information on the methods used to synthesize data from the literature. They used content analysis (*n* = 1),^51^ realist synthesis (*n* = 1),^58^, ^59^ and meta‐analysis (*n* = 2).^50^, ^54^ The software used to perform systems dynamics were: Vensim (*n* = 9),^50^, ^52^, ^53^, ^61^, ^62^, ^63^, ^64^, ^67^, ^68^ and AnyLogic PLE (*n* = 1).^65^

Two reviews using systems dynamics also employed other methods. One review, on technological and economic factors responsible for schedule and cost overrun in research and development projects, used methods for prioritizing and ranking parameters to be included in subsequent models.^61^ Before developing the systems dynamics model, this review used the analytic network process^70^ to calculate a risk priority index of technological and economic factors. Analytic network process was performed using the key factors identified from the literature review and expert opinions. Then, a group of experts judged the weight of each factor and performed pairwise comparisons using the Super Decision software. This allowed identification of the factors that have the highest influence on schedule and cost overrun, which were considered when developing the causal loop diagrams.

Another review interested on skill needs for agri‐food nanotechnology incorporated nonlinear systems dynamics/mixed‐methods modeling. They used qualitative systems analysis to identify system variables and analyze their mutual interactions from data collected in a systematic review, surveys, and interviews with experts and stakeholders.^67^ The authors also performed strategic flexibility analysis to assess the capability of a system to respond to the dynamic environment through changes and actions.^71^ The findings from these methods were used in a final phase to develop causal loop diagrams.

##### Agent‐based modeling

One paper mentioned using agent‐based modeling to understand how the formation and evolution of leisure‐time physical activity population patterns occur.^72^ This method was used to represent the interactions and causal pathways between the agents (i.e., entities of a system) and the environment in a dynamic manner, and to explore the behavior of complex systems. This paper developed a conceptual model to inform the design of agent‐based modeling. Data from the literature review and consultation with experts were used to build a dynamic conceptual model on the psychological and environmental factors influencing leisure‐time physical activity. The next steps planned by the authors include developing and parameterizing the agent‐based model as well as validating the model with real‐world data.^72^

##### Network analysis

One paper conducted a literature review of dynamic modeling studies to identify parameters that impact the progress of construction projects.^73^ They used social network analysis to investigate the interconnectivity among the parameters. This was done by calculating the degree centrality of each parameter, which measures the number of connections one parameter has to the other parameters. The network diagram shows the links and the link strength between the parameters.

##### “Cross‐over” modeling technique

A systematic review was performed to identify theoretical elements and behaviors of asset management data infrastructure and formulated four propositions that were tested in a case study. With the results from both methods, the authors mentioned using a “cross‐over” modeling technique to develop a data model.^69^ It consists of a modeling technique that considers problems from the agent perspective and provides insight into the relationship between agents. The model was built using the Resource Description Framework (RDF), a data model that describes the semantics of information.

#### 3.2.2 Soft system approach

Two reviews mentioned using a soft systems methodology.^74^, ^75^ In Rushton and Lindsay,^74^ a soft systems methodology was used to structure the analysis of the literature on clinical education. They adapted the soft systems methodology and followed three steps: (1) inquiry into situation and analysis, (2) further analysis, and (3) debate and proposed development or change. A content analysis was performed to inform the definition of the different subsystems (e.g., students, supervisor, and tutor) and explore the interactions between the subsystems of the system.

Another review performed a meta‐narrative synthesis of the literature of service quality in information sciences to identify the storylines of the research area.^75^ They used soft systems methodology during the synthesis, which consists of an action‐oriented methodology designed to tackle problematic and messy situations. The review used the CATWOE analysis (Customers, Actors, Transformation process, Worldview, Owners, Environmental constraints) to identify and classify the different elements in each storyline and the links between them. This allowed to develop a rich picture of the storyline of service quality research in which the relationships between the clients, actors, transformations, worldview, owners, and environment (CATWOE) are provided.

#### 3.2.3 Other methods

Another method was identified in one review that used a system of systems framework. Dicks et al.^76^ conducted a review using an inductive grounded theory approach to identify factors influencing the in‐hospital experience of families of potential organ donors and explore relationships within and between factors. During the interpretation of their findings, they used the system of systems theoretical framework. The two systems in their review (i.e., family system and hospital staff system) were broken down into their components and interconnectedness between the component systems were explored. The reviewers developed a systemic map of the psychosocial factors related to potential organ donation using the Kumu systems mapping tool.^76^

### 3.3 Rationales for using a systems methods

Among the papers using systems methods, 10 different reasons for using a systems perspective were identified. The most frequent reason was that systems perspectives allowed authors to understand and account for complexity (*n* = 15).^50^, ^51^, ^52^, ^53^, ^56^, ^60^, ^61^, ^62^, ^64^, ^65^, ^68^, ^69^, ^72^, ^73^, ^74^ In these reviews, they described the complexity of the problem under study and the importance of using systems perspectives to study this complexity. Another common reason was that the systems perspective recognizes the dynamic interaction and synergies between several elements and can simulate interconnected feedback (*n* = 13).^50^, ^52^, ^54^, ^56^, ^57^, ^59^, ^60^, ^62^, ^64^, ^65^, ^67^, ^72^, ^73^ Other reviews justified their use of a systems perspective because it allowed authors to view the system as a whole (provided a holistic view) (*n* = 8).^50^, ^51^, ^57^, ^61^, ^62^, ^68^, ^73^, ^76^ Also, some mentioned it provided a different or new way of thinking compared with the “traditional” way that has some limitations, such as an oversimplification of the problem, assuming a linear process, and not fully grasping impacts of changes (*n* = 2).^73^, ^75^ Other reasons were that systems perspectives account for variations in the degree of influence of stakeholders and unpredictability of their behaviors (*n* = 2),^50^, ^69^ to make predictions by studying different scenarios of behaviors under certain conditions and circumstances over time (*n* = 2),^68^, ^73^ and to help to understand and identify critical factors (*n* = 1).^68^ Also, one review mentioned that the systems approach allowed the reviewers to provide a richer, more nuanced, and truthful representation of a phenomenon.^75^ Finally, papers mentioned using a systems perspective to identify knowledge gaps, and to inform future work such as to guide the prioritization of solutions (*n* = 2),^50^, ^73^ and to improve decision‐making and program development and impact (*n* = 6).^50^, ^52^, ^64^, ^65^, ^67^, ^73^

### 3.4 Limits and challenges of using systems methods

Besides common limits and challenges found in literature reviews such as the paucity of the literature, missing relevant studies, non‐comprehensive literature searches, and high heterogeneity among studies, the included reviews provided eight limits and challenges related to the use of systems methods. First, one challenge often discussed is about the difficulty of capturing the true nature and complexity of the problem under study (*n* = 7).^50^, ^51^, ^52^, ^61^, ^63^, ^68^, ^72^ For example, Kumar and Thakkar^61^ mentioned that “it is difficult to capture the true nature of the decisions and motives for actors' decisions which are dynamic in nature and changes over a period of time.” There is thus a need to update the developed models with time. Also, reviews mentioned that the systems model is a simplified representation of real life (*n* = 3)^53^, ^63^, ^76^ and might be missing elements or links (*n* = 6).^50^, ^51^, ^62^, ^65^, ^72^, ^74^ For example, Adamu et al.^50^ said it is likely that they have missed factors or links in the model since they relied only on published literature. Moreover, some specified that the model did not account for variations from populations, ethnicity, culture, socioeconomic status, or geographic locations/countries (*n* = 3)^53^, ^56^, ^72^ and this can limit the generalizability of the results since they focused on a specific area or population (*n* = 4).^56^, ^61^, ^72^ For example, Garcia et al.^72^ mentioned that most of the evidence found in the literature was primarily from high‐income countries, which can limit the capacity for generalization. Another limitation was that the model was theoretical (based on literature only) and needed to be empirically tested and calibrated (*n* = 5).^51^, ^60^, ^63^, ^64^, ^68^ Moreover, some mentioned they did not perform weighting, adjustment, or subgroup analysis based on some characteristics of included studies (*n* = 4).^50^, ^55^, ^68^, ^72^ For example, Frerichs et al.^56^ said that they reviewed studies with a range of research designs and sample sizes, and they did not rank or weigh evidence according to design strength or assess the magnitude of effect sizes. Finally, some reviews discussed the influence of the authors' experience and preferences on the choice of the approach and parameters used, and their implication on the model found (*n* = 3).^50^, ^52^, ^67^

## 4 DISCUSSION

Systems perspectives have been growing in popularity in evidence synthesis over the past decade.^6^, ^77^, ^78^ In this methodological review, 98 reviews adopting a systems perspective were analyzed. These reviews addressed different aims such as to conceptualize a phenomenon, identify and understand risk factors, and examine the process and outcomes of an intervention (see Files S1 and S2). Two main categories of papers were identified: reviews using a systems lens, and reviews using systems methods. The main reasons for using systems perspectives were to address complexity, view the problem as a whole, and understand the interrelationships between the elements. Adopting this way of thinking can open the doors to a variety of methods and methodologies that focus on adopting a more holistic perspective and considering the complex nature of a system. Some papers, however, discussed the challenges of capturing the true nature and complexity of a problem and potential limits of these methods used, such as the risk of missing factors and interconnections, and not accounting for population or contextual variations.

Beside from using a systems lens to guide a review, this methodological review also identified a variety of methods to analyze data. Evidence synthesis researchers might be familiar with some of the methods used such as thematic synthesis, realist synthesis, framework synthesis, and meta‐analysis. However, several systems methods were identified that may be new to evidence synthesis researchers, such as systems dynamics, agent‐based modeling, and soft systems methodology (Table 4). They enable many aspects to be considered within a system by focusing on the linkages, interrelationships, interactions, and behaviors among the elements that characterize a whole system.^79^, ^80^ Also, when using a systems perspective, the questions addressed can be different. For example, in the literature on systems thinking, researchers have asked questions such as *How do the elements interact with each other to form whole systems*? *How do boundary distinctions define systems*? and *How do the different perspectives influence the interrelationships, systems, and boundary distinctions within a system*?^19^ Moreover, the elements studied can be different. For example, Higgins et al.^78^ have suggested a variety of synthesis possibilities to address the different attributes of complex systems in systematic reviews such as using meta‐regression to consider the interactions between the intervention and context, the system adaptivity, and the emergent properties. Thus, when adopting a systems perspective, there is a shift of focus from specific outcomes and the different components of an intervention toward the different attributes of a complex system (Table 1).

Three main types of systems approaches have been proposed in the literature (hard, soft, and critical; see Table 2). In this methodological mapping review, the majority of included reviews used a hard systems approach, two reviews mentioned using soft systems approach, and none adopted a critical systems approach. Although there are epistemological differences between these three approaches, when applied to reviews, several similarities could be found. In general, regardless of the approach used, the findings from reviews were useful to define the problem, understand concepts, identify and define key elements, investigate and understand relationships between the elements, develop an initial systems model, and/or identify research gaps. The main difference between the approaches is in the methods used and interpretation of the findings. In this methodological mapping review, a variety of quantitative and qualitative methods as well as different combinations of methods were identified for tackling complex situations. In the reviews that used a soft systems approach,^74^, ^75^ the methods were predominantly qualitative such as meta‐narrative synthesis and content analysis. In the hard systems approach, although modeling systems methods are predominantly quantitative, they can also involve qualitative methods. For instance, the first steps of systems dynamics consist of creating initial models such as causal loop diagrams and stock‐and‐flow diagrams, and are considered “qualitative” systems dynamics since they do not involve computer simulation modeling.^81^ These steps can involve identifying and selecting systems variables, and performing qualitative analysis of the interaction among the variables. In the last steps of systems dynamics, quantitative methods could be more important since they consist of testing and calibrating the model using simulation modeling and performing sensitivity analysis. In addition, these steps require to go beyond the conventional systematic review, and incorporate a wider range of evidence in the synthesis.

The examples of reviews using systems methods presented often had two characteristics in common: first, the reviews contained multiple processes, often involving the development of a conceptual model, followed by more quantitative analysis. Second, the reviews utilized a wide range of types of research to inform the development of models, and the specification of parameter values. Neither characteristic is usually part of a conventional systematic review, where usually a specific question is addressed using a relatively homogenous set of included studies. Thus, it would appear that the use of a systems method is not something that can be easily added into a conventional systematic review. It rather requires detailed upfront planning which should include an appraisal of team skills and timelines. The methods described above often require expertise that is not always present in systematic review teams. Also, the multicomponent nature of the review process will probably require longer timelines than conventional systematic reviews need.

### 4.1 Limitations

Some limitations of this methodological review need to be acknowledged. The search was limited to papers published in English or in French found in seven databases and three search engines. Also, the search strategy included a range of terms related with “systems perspective.” A search term on “systems dynamic” was included in the search strategy to refer to one of the characteristics of complex systems (see Table 1). As seen in the results, “systems dynamic” can also refer to a method. Some individual named methods were not part of the search strategy (e.g., soft systems methodology, critical systems approach), which may mean they were less likely to be identified if they exist. Moreover, although some non‐discipline‐specific sources were searched, evidence synthesis having adopted a systems perspective in disciplines not indexed in the sources may have been missed. Finally, there was no duplicate eligibility assessment and data extraction. Another reviewer was involved in 5% of the screening and data extraction to help clarify the eligibility criteria and data to extract.

## 5 CONCLUSION

Using a systems perspective offers a way of understanding complex systems and can be particularly relevant when addressing complex situations. This methodological mapping review has identified a variety of methods that were used to understand complex dynamic interactions between various elements within a whole system. These methods provide different analytical possibilities from existing methods for addressing complexity when applied to evidence synthesis. Also, reviews can combine a diversity of evidence to provide a broad and more complete picture of the literature of a complex system. These approaches can enhance the applicability of findings from evidence synthesis for patients, stakeholders, and decision‐makers by taking account of context and thinking more broadly in terms of the interaction of the different elements within a system.^77^

The results of this methodological mapping review provide a first step to understand how systems perspectives can be used in evidence synthesis. It may be useful for helping to define what we mean by a “systems perspective evidence synthesis”; identifying the conditions under which it might be useful to take this approach; and giving reviewers a starting point when designing and planning a systems perspective evidence synthesis. More methodological development is still needed to provide guidance on how to address systems complexity in evidence synthesis, which systems methods are appropriate in which circumstances, and how to integrate the different data and levels of research. The challenges for using systems perspectives are numerous, requiring a change in the way one sees and thinks about the world. There is a need to develop new knowledge and skills to implement these approaches. Also, there is a need to establish collaborations between systems researchers and evidence synthesis researchers, and to reflect on what different disciplines and methodologies can learn from each other to improve the development of research on complexity.

## AUTHOR CONTRIBUTIONS

QNH and JT conceived and designed the review. QNH was involved in all the steps of the review with the collaboration of CS for the search, MB for the selection and data extraction, and DK, AOM and LvG for the synthesis. All authors critically revised and approved the manuscript.

## Supporting information


**Appendix S1 and S2** Supporting information

## Data Availability

Data sharing is not applicable to this article as no new data were created or analyzed in this study.

## References

[1] Guise J‐M , Chang C , Butler M , Viswanathan M , Tugwell P . AHRQ series on complex intervention systematic reviews—paper 1: an introduction to a series of articles that provide guidance and tools for reviews of complex interventions. J Clin Epidemiol. 2017;90:6‐10. 28720511 10.1016/j.jclinepi.2017.06.011

[2] Thomas J , Petticrew M , Noyes J , et al. Chapter 17: intervention complexity. In: Higgins JPT , Thomas J , Chandler J , et al., eds. Cochrane Handbook for Systematic Reviews of Intervention. John Wiley & Sons; 2019.

[3] Caldwell DM . An overview of conducting systematic reviews with network meta‐analysis. Syst Rev. 2014;3:109. 25267336 10.1186/2046-4053-3-109 PMC4183945

[4] Kneale D , Thomas J , Bangpan M , Waddington H , Gough D . Conceptualising causal pathways in systematic reviews of international development interventions through adopting a causal chain analysis approach. J Dev Effect. 2018;4:1‐16.

[5] Langlois ÉV , Daniels K , Akl EA , World Health Organization . Evidence Synthesis for Health Policy and Systems: a Methods Guide. World Health Organization; 2018 http://www.who.int/alliance-hpsr/resources/publications/Alliance-evidence-synthesis-MethodsGuide.pdf 33877749

[6] Petticrew M , Knai C , Thomas J , et al. Implications of a complexity perspective for systematic reviews and guideline development in health decision making. BMJ Glob Health. 2019;4(Suppl 1):e000899. 10.1136/bmjgh-2018-000899 PMC6350708 30775017

[7] Norris SL , Rehfuess EA , Smith H , et al. Complex health interventions in complex systems: improving the process and methods for evidence‐informed health decisions. BMJ Glob Health. 2019;4(Suppl 1):e000963. 10.1136/bmjgh-2018-000963 PMC6350736 30775018

[8] Noyes J , Booth A , Moore G , Flemming K , Tunçalp Ö , Shakibazadeh E . Synthesising quantitative and qualitative evidence to inform guidelines on complex interventions: clarifying the purposes, designs and outlining some methods. BMJ Glob Health. 2019;4(Suppl 1):e000893. 10.1136/bmjgh-2018-000893 PMC6350750 30775016

[9] Meadows DH . Thinking in Systems: A Primer. Sustainability Institute; 2008.

[10] Brainard J , Hunter PR . Do complexity‐informed health interventions work? A scoping review. Implement Sc. 2016;11(1):127. 27647152 10.1186/s13012-016-0492-5 PMC5029105

[11] Ellis BS , Herbert S . Complex adaptive systems (CAS): an overview of key elements, characteristics and application to management theory. J Innov Health Inform. 2010;19(1):33‐37. 10.14236/jhi.v19i1.791 22118334

[12] Northridge ME , Metcalf SS . Enhancing implementation science by applying best principles of systems science. Health Res Policy Syst. 2016;14(1):74. 27716275 10.1186/s12961-016-0146-8 PMC5050576

[13] Rutter H , Savona N , Glonti K , et al. The need for a complex systems model of evidence for public health. Lancet. 2017;390(10112):2602‐2604. 28622953 10.1016/S0140-6736(17)31267-9

[14] Yawson RM . Systems theory and thinking as a foundational theory in human resource development‐a myth or reality? Hum Resour Dev Rev. 2013;12(1):53‐85.

[15] Reynolds M . Critical thinking and systems thinking: towards a critical literacy for systems thinking in practice. In: Horvath CP , Forte JM , eds. Critical Thinking. Nova Science Publishers, Inc.; 2011:37‐48.

[16] Plsek P , Greenhalgh T . The challenge of complexity in health care: an introduction. BMJ. 2001;323(7314):625‐628. 11557716 10.1136/bmj.323.7313.625 PMC1121189

[17] Braithwaite J . Changing how we think about healthcare improvement. BMJ. 2018;361:k2014. 29773537 10.1136/bmj.k2014 PMC5956926

[18] Viale R , Pozzali A . Complex adaptive systems and the evolutionary triple helix. Crit Sociol. 2010;36(4):575‐594.

[19] Midgley G , Rajagopalan R . Critical systems thinking, systemic intervention and beyond. The Handbook of Systems Science. Springer; 2019.

[20] Hummelbrunner R . Systems thinking and evaluation. Evaluation. 2011;17(4):395‐403.

[21] Watson SL , Watson WR . Critical systems theory for qualitative research methodology. In: Dennis B , Carspecken L , Carspecken PF , eds. Qualitative Research: A Reader in Philosophy, Core Concepts, and Practice. Peter Lang; 2013:111‐127.

[22] Jackson MC . The origins and nature of critical systems thinking. Syst Practice. 1991;4(2):131‐149.

[23] Carey G , Malbon E , Carey N , Joyce A , Crammond B , Carey A . Systems science and systems thinking for public health: a systematic review of the field. BMJ Open. 2015;5(12):e009002. 10.1136/bmjopen-2015-009002 PMC4710830 26719314

[24] Scherer HH , Holder L , Herbert B . Student learning of complex earth systems: conceptual frameworks of earth systems and instructional design. J Goesci Educ. 2017;65(4):473‐489.

[25] Williams A , Kennedy S , Philipp F , Whiteman G . Systems thinking: a review of sustainability management research. J Clean Prod. 2017;148:866‐881.

[26] Lawson DO , Leenus A , Mbuagbaw L . Mapping the nomenclature, methodology, and reporting of studies that review methods: a pilot methodological review. Pilot Feasibility Stud. 2020;6(1):13. 32699641 10.1186/s40814-019-0544-0 PMC7003412

[27] James KL , Randall NP , Haddaway NR . A methodology for systematic mapping in environmental sciences. Envrion Evid. 2016;5(1):7.

[28] Oakley A , Gough D , Oliver S , Thomas J . The politics of evidence and methodology: lessons from the EPPI‐Centre. Evid Policy. 2005;1(1):5‐32.

[29] Rusoja E , Haynie D , Sievers J , et al. Thinking about complexity in health: a systematic review of the key systems thinking and complexity ideas in health. J Eval Clin Pract. 2018;24(3):600‐606. 29380477 10.1111/jep.12856

[30] Sturmberg JP , Martin CM , Katerndahl DA . Systems and complexity thinking in the general practice literature: an integrative, historical narrative review. Ann Fam Med. 2014;12(1):66‐74. 24445105 10.1370/afm.1593 PMC3896540

[31] Wilkinson J , Goff M , Rusoja E , Hanson C , Swanson RC . The application of systems thinking concepts, methods, and tools to global health practices: an analysis of case studies. J Eval Clin Pract. 2018;24(3):607‐618. 29152819 10.1111/jep.12842

[32] Thomas J , Graziosi S , Brunton J , et al. EPPI‐Reviewer: Advanced Software for Systematic Reviews, Maps and Evidence Synthesis. EPPI‐Centre, UCL Social Research Institute, University College London; 2020.

[33] Zali MR , Najafian M , Colabi AM . System dynamics modeling in entrepreneurship research: a review of the literature. Int J Supply Oper Manag. 2014;1(3):347‐370.

[34] Evans D . Systematic reviews of interpretive research: interpretive data synthesis of processed data. Aust J Adv Nurs. 2002;20(2):22‐26. 12537149

[35] Hong JS , Huang H , Sabri B , Kim JS . Substance abuse among Asian American youth: an ecological review of the literature. Child Youth Serv Rev. 2011;33(5):669‐677.

[36] Sahin E , Vidal LA , Benzarti E . A framework to evaluate the complexity of home care services. Kybernetes. 2013;42(4):569‐592.

[37] Phillips AB , Merrill JA . Innovative use of the integrative review to evaluate evidence of technology transformation in healthcare. J Biomed Inform. 2015;58:114‐121. 26429591 10.1016/j.jbi.2015.09.014 PMC4684715

[38] Kusuwo P , Myezwa H , Pilusa S , M'Kumbuzi V . A systematic review to identify system‐related elements that can be used to evaluate community‐based rehabilitation (CBR) programmes. Eur J Phys. 2017;19(Supplement 1):41‐46.

[39] Aksulu A , Wade M . A comprehensive review and synthesis of open source research. J Assoc Inf Syst. 2010;11(11):576‐656.

[40] Holloway Cripps KG . *Too Hot, Too Cold, and Then Just Right: Balancing Form and Function in Biophilic Designed Office Workspaces*. Doctoral dissertation. University of Maryland University College; 2016.

[41] McLain R , Lawry S , Ojanen M . Fisheries' property regimes and environmental outcomes: a realist synthesis review. World Dev. 2018;102:213‐227.

[42] Reid SA , Rodney A , Kama M , Hill PS . A process for developing multisectoral strategies for zoonoses: the case of leptospirosis in Fiji. BMC Public Health. 2017;17(1):671. 28830472 10.1186/s12889-017-4673-1 PMC5567884

[43] Lorthios‐Guilledroit A , Richard L , Filiatrault J . Factors associated with the implementation of community‐based peer‐led health promotion programs: a scoping review. Eval Program Plann. 2018;68:19‐33. 29459228 10.1016/j.evalprogplan.2018.01.008

[44] Noyes J , Brenner M , Fox P , Guerin A . Reconceptualizing children's complex discharge with health systems theory: novel integrative review with embedded expert consultation and theory development. J Adv Nurs. 2014;70(5):975‐996. 24164460 10.1111/jan.12278

[45] Hirano KA , Rowe D , Lindstrom L , Chan P . Systemic barriers to family involvement in transition planning for youth with disabilities: a qualitative metasynthesis. J Child Fam Stud. 2018;27:3440‐3456.

[46] Strom KJ , Martin AD , Villegas AM . Clinging to the edge of chaos: the emergence of practice in the first year of teaching. Teach Coll Rec. 2018;120(7):1‐32.

[47] Shigayeva A , Coker RJ . Communicable disease control programmes and health systems: an analytical approach to sustainability. Health Policy Plan. 2015;30(3):368‐385. 24561988 10.1093/heapol/czu005

[48] Butt G , Markle‐Reid M , Browne G . Interprofessional partnerships in chronic illness care: a conceptual model for measuring partnership effectiveness. Int J Integr Care. 2008;8:1‐14. 10.5334/ijic.235 PMC2387190 18493591

[49] Techera U , Hallowell M , Stambaugh N , Littlejohn R . Causes and consequences of occupational fatigue: meta‐analysis and systems model. J Occup Environ Med. 2016;58(10):961‐973. 27525527 10.1097/JOM.0000000000000837

[50] Adamu AA , Sarki AM , Uthman OA , Wiyeh AB , Gadanya MA , Wiysonge CS . Prevalence and dynamics of missed opportunities for vaccination among children in Africa: applying systems thinking in a systematic review and meta‐analysis of observational studies. Expert Rev Vaccines. 2019;18(5):547‐558. 30822248 10.1080/14760584.2019.1588728

[51] Azevedo BB , Saurin TA . Losses in water distribution systems: a complexity theory perspective. Water Resour Manag. 2018;32(9):2919‐2936.

[52] Brzezina N , Kopainsky B , Mathijs E . Can organic farming reduce vulnerabilities and enhance the resilience of the european food system? A critical assessment using system dynamics structural thinking tools. Sustainability. 2016;8(10):32.

[53] Chidumayo NN . System dynamics modelling approach to explore the effect of dog demography on rabies vaccination coverage in Africa. PLoS One. 2018;13(10):e0205884. 30359399 10.1371/journal.pone.0205884 PMC6201891

[54] Eickhoff SB , Heim S , Zilles K , Amunts K . A systems perspective on the effective connectivity of overt speech production. Phil Trans R Soc A. 2009;367(1896):2399‐2421. 19414462 10.1098/rsta.2008.0287 PMC3268212

[55] Frerichs L. *Architecture and Design for Healthy Eating in Schools*. Doctoral dissertation. University of Nebraska; 2015.

[56] Frerichs L , Brittin J , Sorensen D , et al. Influence of school architecture and design on healthy eating: a review of the evidence. Am J Public Health. 2015;105(4):e46‐e57. 10.2105/AJPH.2014.302453 PMC4358206 25713964

[57] Guo S , Roudsari A , Garcez A . A causal loop approach to the study of diagnostic errors. Stud Health Technol Inform. 2014;205:73‐77. 25160148

[58] Hagg H. *Large System Transformation within Healthcare Organizations Utilizing Lean Deployment Strategies*. Doctoral dissertation. Worcester Polytechnic Institute; 2013.

[59] Woodward‐Hagg H , Bar‐On I . Large system transformation within healthcare organizations utilizing lean deployment strategies. Paper presented at: IIE Annual Conference Proceedings; 2014:458–467.

[60] Jalali SMJ . *Three Essays on Systems Thinking and Dynamic Modeling in Obesity Prevention Interventions*. Doctoral dissertation. Virginia Polytechnic Institute and State University; 2015.

[61] Kumar S , Thakkar JJ . Schedule and cost overrun analysis for R&D projects using ANP and system dynamics. Int J Qual Reliab Manage. 2017;34(9):1551‐1567.

[62] Martinez Garcia D , Sheehan MC . Extreme weather‐driven disasters and Children's health. Int J Health Serv. 2016;46(1):79‐105. 26721564 10.1177/0020731415625254

[63] Oesterreich TD , Teuteberg F . Why one big picture is worth a thousand numbers: measuring intangible benefits of investments in augmented reality based assistive technology using utility effect chains and system dynamics. Inf Syst Manag. 2018;16(2):407‐441.

[64] Schuh HB , Merritt MW , Igusa T , Lee BY , Peters DH . Examining the structure and behavior of Afghanistan's routine childhood immunization system using system dynamics modeling. Int J Health Gov. 2017;22(3):212‐227.

[65] Xie T , Liu W , Anderson BD , Liu X , Gray GC . A system dynamics approach to understanding the one health concept. PLoS One. 2017;12(9):e0184430. 28877267 10.1371/journal.pone.0184430 PMC5587294

[66] Yawson RM . Systematic review to identify skill needs for agrifood nanotechnology workforce. Career Tech Educ Res. 2017;42(3):149‐181.

[67] Yawson RM , Greiman BC . A systems approach to identify skill needs for agrifood nanotechnology: a multiphase mixed methods study. Hum Resour Dev Q. 2016;27(4):517‐545.

[68] Zammar G , Meister H , Shah J , Phadtare A , Cofiel L , Pietrobon R . So different, yet so similar: meta‐analysis and policy modeling of willingness to participate in clinical trials among Brazilians and Indians. PLoS One. 2010;5(12):e14368. 21179556 10.1371/journal.pone.0014368 PMC3002940

[69] Brous P , Janssen M , Herder P . Next generation data infrastructures: towards an extendable model of the asset management data infrastructure as complex adaptive system. Complexity. 2019;2019:1‐17.

[70] Saaty TL , Vargas LG . Decision Making with the Analytic Network Process. Springer; 2006.

[71] Yawson R . Strategic flexibility analysis of HRD research and practice post COVID‐19 pandemic. Hum Resour Dev Int. 2020;23:406‐417.

[72] Garcia LMT , Roux AVD , Martins ACR , Yang Y , Florindo AA . Development of a dynamic framework to explain population patterns of leisure‐time physical activity through agent‐based modeling. Int J Behav Nutr Phys Act. 2017;14:111. 28830527 10.1186/s12966-017-0553-4 PMC5568398

[73] Abotaleb IS , El‐adaway IH . Managing construction projects through dynamic modeling: reviewing the existing body of knowledge and deriving future research directions. J Manag Eng. 2018;34(6):04018033.

[74] Rushton A , Lindsay G . Clinical education: a critical analysis using soft systems methodology. Int J Ther Rehabil. 2003;10(6):271‐280.

[75] Sylvester A , Tate M , Johnstone D . Beyond synthesis: re‐presenting heterogeneous research literature. Behav Inform Technol. 2013;32(12):1199‐1215.

[76] Dicks SG , Ranse K , van Haren FM , Boer DP . In‐hospital experiences of families of potential organ donors: a systematic review and qualitative synthesis. Health Psychol Open. 2017;4(2):2055102917709375. 28680696 10.1177/2055102917709375 PMC5444581

[77] Noyes J , Gough D , Lewin S , et al. A research and development agenda for systematic reviews that ask complex questions about complex interventions. J Clin Epidemiol. 2013;66(11):1262‐1270. 23953084 10.1016/j.jclinepi.2013.07.003

[78] Higgins JP , López‐López JA , Becker BJ , et al. Synthesising quantitative evidence in systematic reviews of complex health interventions. BMJ Glob Health. 2019;4(Suppl 1):e000858. 10.1136/bmjgh-2018-000858 PMC6350707 30775014

[79] Cabrera D , Colosi L , Lobdell C . Systems thinking. Eval Program Plann. 2008;31(3):299‐310. 18272224 10.1016/j.evalprogplan.2007.12.001

[80] De Savigny D , Adam T . Systems Thinking for Health Systems Strengthening. Alliance for Health Policy and Systems Research and World Health Organization; 2009.

[81] Wolstenholme EF . Qualitative vs quantitative modelling: The evolving balance. J Oper Res Soc. 1999;50(4):422‐428.

[82] Penney LS , Nahid M , Leykum LK , et al. Interventions to reduce readmissions: can complex adaptive system theory explain the heterogeneity in effectiveness? A systematic review. BMC Health Serv Res. 2018;18(1):894. 30477576 10.1186/s12913-018-3712-7 PMC6260570

[83] Richardson KA , Cilliers P , Lissack M . Complexity science: a "gray" science for the" stuff in between". Emergence. 2001;3(2):6‐18.

[84] Luke DA , Stamatakis KA . Systems science methods in public health: dynamics, networks, and agents. Annu Rev Public Health. 2012;33:357‐376. 22224885 10.1146/annurev-publhealth-031210-101222 PMC3644212

[85] Wiek A , Lang DJ , Siegrist M . Qualitative system analysis as a means for sustainable governance of emerging technologies: the case of nanotechnology. J Clean Prod. 2008;16(8–9):988‐999.

[86] Checkland P , Poulter J . Soft systems methodology. In: Reynolds M , Holwell S , eds. Systems Approaches to Making Change: A Practical Guide. Springer; 2020:201‐253.

[87] Haimes YY . Modeling complex systems of systems with phantom system models. Syst Eng. 2012;15(3):333‐346.

