# Supplementary material for: Using systems perspectives in evidence synthesis: A methodological mapping review
Source: Res Synth Methods. 2022 Aug 18;13(6):667–80. doi: 10.1002/jrsm.1595 (PMC9804835; doi:10.1002/jrsm.1595)
Supplement: Supplementary file 1 — Appendix S1 and S2 Supporting information [file JRSM-13-667-s001.docx]

**Supplementary file 1.** Characteristics of Included Papers using systems lens and/or developing systems models (n=76)

| **Authors**  **(year)** | **Country of lead author** | **Field** | **Aim** | **Number included papers** | **Sytems theory** | **Synthesis methods reported** | **Systems model developed** |
| --- | --- | --- | --- | --- | --- | --- | --- |
| Aksulu and Wade (2010) | UK | Information sciences | Present a comprehensive taxonomy of existing open source research, to develop a framework to organize this research, and to chart a course for future work. | 618 | General systems theory | Multistage content anlaysis process | - |
| Alexander et al. (2014) | UK | Organizational studies | Examine the use of decision theory concepts in sustainable supply chain management research. | 160 | Cynefin framework | Abductive approach | - |
| Basurto et al. (2013) | USA | Environmental studies | Diagnose what combinations of social–ecological system variables were associated with fishers’ ability to self-organize and avoid overexploiting their fisheries, and which interactions led to continued overharvesting. | - | Ostrom’s social–ecological system  framework | - | - |
| Benton et al. (2013) | Switzerland | Health | Explore whether the seven component concepts of the open systems framework can be used to analyze and document existing literature on regulatory trends. | 64 | Open systems theory | - | Open to environment |
| Best et al. (2012) | Canada | Health | Analyze examples of successful and less successful transformation initiatives, to synthesize knowledge of the underlying mechanisms, to clarify the role of government, and to outline options for evaluation. | 84 | Complex adaptive system theory | Realist synthesis | - |
| Bowen et al. (2015) | USA | Health | Examine the process and outcomes of nutrition environment interventions. | - | Socioecological framework | Qualitative synthesis | - |
| Burns et al. (2015) | USA | Health | Examine the frequency with which research published in School Psychology Review over the past 10 years incorporated an ecological systems framework. | 349 | Ecological systems theory | - | - |
| Butt et al. (2008) | Canada | Health | Conceptualize interprofessional health and social service partnerships at the front-line staff level, and identify tools valid for group process measurement. | - | Complexity theory | Thematic synthesis | Conceptual model of the attributes of interprofessional collaborative processes |
| Cameron et al. (2018) | Canada | Health | Examine the etiology of borderline personality disorder through a biopsychosocial framework. | 18 | Ecological systems theory | - | - |
| Cassarino and Murphy (2018) | Ireland | Health | Identify the most important indicators of crash risk in young novice drivers, considering individual, social, and environmental circumstances and highlight the most effective preventive factors for young drivers. | 196 | Ecological systems theory | - |  |
| Dallat et al. (2017) | Australia | Health | Review the risk assessment methods and evaluate the extent to which they are underpinned by a systems thinking approach. | 342 | Rasmussen’s Risk Management Framework | - | - |
| Davidson (1997) (dissertation) | USA | Organizational studies | Develop a values-based system model for organizational change. | - | General system theory | - | Systems model |
| Dresch et al. (2018) | Brazil | Organizational studies | Understand the concept of competitiveness at firm level and its relation to productivity. | 54 | Systems thinking | - | Systemic map |
| Duckworth and Azulay Cbertok (2012) | USA | Health | Examine the literature on smoking cessation interventions targeted at pregnant women’s partners during pregnancy and early postpartum. | 5 | Family system theory | - | - |
| Eklund and Salzmann-Erikson (2016) | Sweden | Health | Describe how eating disorders among adolescents affect family relationships and the family’s daily living conditions and to describe the family’s experienced need for professional support. | 15 | Family system theory | Data reduction, data display, data compassion and conclusion drawing using mind-map and verification. | - |
| El-Behadli et al. (2015) | USA | Health | Extend the UNICEF care model to focus on childhood obesity and its associated risks with an emphasis on the emotional climate of the parent–child relationship within the family. | 16 | Systems approach | - | Adaptation of the UNICEF care model |
| Elnitsky et al. (2017) | USA | Health | Synthesize evidence on military service members and veterans reintegration and adapt a social ecological systems model. | 186 | Ecological systems theory | Comparative analysis | - |
| Fulgencio et al. (2016) | Netherlands | Organizational studies | Study how has organizational social value been scholarly or academically analyzed in the field of business and economics. | 45 | Ecological systems theory and Input, process, output and environment (IPOE) framework | Scientometric analysis and mapping | - |
| Furst et al. (2019) | Australia | Health | Review the methods of context analysis of mental health systems. | 46 | Systems dynamics complexity approach | - | - |
| Gissi et al. (2019) | Italy | Environmental studies | Detect future research needs to support marine/maritime spatial planning (MSP) practices that account for system dynamics and future change, and to provide methods for incorporating change and dynamics in MSP processes. | 202 | Ecological systems theory | - | - |
| Goode et al. (2019) | Australia | Health | Synthesis and summarise the evidence relating to the risk factors associated with musculoskeletal disorders in the workplace. | 31 | Rasmussen’s Risk Management Framework | Classification | - |
| Greenlee et al. (2018) | USA | Health | Determine the role of family level processes on outcomes for those with autism spectrum disorder (ASD) from a family system perspective and whether and how culture has been integrated into discussion of family processes in ASD families. | 9 | Family systems theory | - | - |
| Hamidi (2018) | Malaysia | Organizational studies | Investigate what is known in the literature about human resource development in developing economies’ in small - medium enterprises. | 28 | Systems theory | - | - |
| Hirano et al. (2018) | USA | Child and family studies | Synthesize and make meaning from individual qualitative studies exploring the lived experience of parents and caregivers of young adults with disabilities in the process of planning and preparing for their youth’s transition from high school to adult roles. | 23 | Ecological systems theory | Meta-synthesis method | - |
| Holdsworth et al. (2017) | UK | Physical activity | Map a systems-based framework of the factors influencing dietary and physical activity behaviours in ethnic minority populations living in Europe. | - | Systems thinking | Concept mapping | Systems-based framework of the priority factors and clusters influencing dietary behaviours |
| Holloway Cripps (2016)  (dissertation) | USA | Organizational studies | Identify the effects of biophilic design on office employees in the workspace. | 31 | Systems theory | Realist synthesis | Updated Conceptual Model |
| Hong et al. (2011) | USA | Child and family studies | Understand the risk and protective factors that are associated with substance use among Asian American youth. | 24 | Ecological systems theory | - | - |
| Hong et al. (2012) | USA | Child and family studies | Review studies on the risk and protective factors for parent-directed youth aggression and frame the findings within the contexts of sociodemographic characteristics and the ecological systems framework. | 30 | Ecological systems theory | - | - |
| Jackson et al. (2017) | UK | Child and family studies | Synthesize the prospective longitudinal research findings on the protective factors against extrafamilial victimization. | 13 | Ecological systems theory | Narrative data synthesis | - |
| Javadi et al. (2017) | Switzerland | Health | Determine whether a systems approach is employed in the implementation and evaluation of task shifting for mental health using lay providers in low- and middle-income countries, and to highlight system-wide effects of task-shifting strategies. | 30 | Systems thinking | - | - |
| Kennedy et al. (2019) | USA | Child and family studies | Understand the environmental impact of youth inquiry approaches. | 68 | Ecological systems theory | - | - |
| Knocke and Schuster (2017) | Germany | Organizational studies | Analyze where and when repatriation research has been published, what aspects have been considered in the repatriation literature, and how the research has been conducted. | 96 | Ecological systems theory | Content-related analysis | Layers of the ecological systems theory |
| Kuch (2017) | Germany | Environmental studies | Understand the relation  between proximity and ecological sustainability from a spatial perspective. | 63 | Systems theory | Categorization | - |
| Kuhnen and Hahn (2018) | Germany | Organizational studies | Evaluate research on social indicators from an open systems theory perspective and provide a conceptual synthesis that may guide future research. | 141 | Open systems theory | Abductive analysis | - |
| Kupers et al. (2019) | Netherlands | Education | Integrate the main theoretical approaches and empirical research to defining and understanding children’s creativity. | 184 | Complex dynamic systems theory | - | Complex dynamic systems model of creativity |
| Kusuwo et al. (2017) | South Africa | Health | Determine the types of elements used in community-based rehabilitation (CBR) evaluation literature and to document the system-related elements that were used to evaluate CBR programmes. | 12 | Systems thinking | Inductive content analysis | - |
| Lapalme et al. (2014) | Canada | Child and family studies | Explore how neighbourhood interventions promote positive youth development and the role of context for these interventions. | 172 | Ecological systems theory | Narrative | - |
| Leykum et al. (2007) | USA | Health | Examine the relationship between interventions that leverage complex adaptive systems characteristics in intervention design and implementation, and effectiveness of reported outcomes for patients with Type II diabetes. | 32 | Complex adaptive systems framework | Rating and Fisher's exact test | - |
| Leykum et al. (2010) | USA | Health | Examine whether interventions to improve outcomes of patients with congestive heart failure consistent with complex adaptive systems are more likely to be effective. Explore the potential impact of the nature of the disease on the types of interventions that are more likely to be effective. | 44 | Complex adaptive systems framework | Rating and Fisher's exact test | - |
| Liao et al. (2011) | USA | Child and family studies | Examine factors that contribute to child maltreatment in China. | 24 | Ecological systems theory | - | - |
| Lorthios-Guilledroit et al. (2018) | Canada | Health | Describe the factors likely to be associated with implementation of community-based peer led health promotion programs for adults. | 55 | Complex adaptive systems perspective | Best fit framework synthesis  Content analysis | Peer-led health promotion programs guiding framework |
| MacDonald et al. (2012b) | Canada | Health | Understand how leaders of nursing associations set priorities and take action for healthy public policies. | 40 | Socioecological systems perspective | Descriptive-analytical narrative method | - |
| MacDonald et al. (2012a) | Canada | Health | Identify research that explored or explained how nursing associations were engaged in community environmental health policy setting and advocacy. | - | Socioecological systems perspective | - | Conceptual framework |
| Marek and Wu (2014) | USA | Education | Identify a typology of internal and external environmental factors that influence successful instructional design for computer assisted language learning. | - | Chaos theory  Complex dynamic systems | Compare/ contrast and grouping of factors | Ecology model |
| Marheineke et al. (2016) | Germany | Organizational studies | Understand the impact of boundary objects on the virtual collaboration process. | 175 | Complex socio-technical systems | - | - |
| McLain et al. (2018) | USA | Environmental studies | Synthesizing evidence on environmental outcomes of marine protected areas governed under different types of property regimes. | 31 | Socioecological framework | Realist synthesis | - |
| Meade and Ickovics (2005) | USA | Health | Document rates of sexually transmitted diseases, repeat pregnancy, condom use, and contraception among pregnant/mothering teens; identify correlates of these biological and behavioral outcomes; and review sexual risk reduction interventions. | 51 | Ecological systems theory | - | Adapted ecological systems model of sexual risk |
| Merali et al. (2012) | UK | Information sciences | Develop a meta-level systemic perspective of the dimensions of change in the strategic information systems (SIS) domain as it accommodates the changes in information system research and practice over time. | 170 | Complex adaptive systems | - | - |
| Miles et al. (2019) | USA | Child and family studies | Review the experiences associated with perimigration and their impact on caregivers and children from an ecological systems perspective. | 34 | Ecological systems theory | - | - |
| Minh et al. (2017) | Canada | Child and family studies | Synthesize evidence on the structural determinants, mediators, and moderators of the relationship between neighborhoods and developmental health, and to summarize current evidence on how, where, and for whom neighborhoods matter in early childhood. | 42 | Ecological systems theory | Descriptive | - |
| Mitchell et al. (2003) | Australia | Health | Identify (a) a conceptual framework which explains the effect of critical illness on families; (b) the concept of ‘uncertainty in illness’ and anxiety as it may apply to these families and; (c) their needs, particularly about transfer from ICU. | 17 | General systems theory | - | - |
| Newig and Fritsch (2009) | Germany | Environmental studies | Test the hypothesis that certain participatory processes and analytical decision tools are particularly useful for improving multi-level environmental governance. | 47 | Complex adaptive systems | Case survey  Spearman’s rho | - |
| Noyes et al. (2014) | UK | Health | Identify critical components in the 5 level health system, and the barriers and facilitators, and critical success factors, to transition from hospital to home of children with complex healthcare needs. | 34 | Health systems theory | Framework synthesis | Five Level Health System Model |
| Odom et al. (2004) | USA | Education | Summarize the state of knowledge about preschool inclusion emerging primarily from the U.S. literature and to determine the implications of this knowledge for practice. | - | Ecological systems theory | - | - |
| Okwir et al. (2018) | Sweden | Organizational studies | Understand the emergence of complexities while implementing and using performance management systems in organizations. | 76 | Complexity theory | Synthesis, pattern-matching and integration | - |
| Penney et al. (2018) | USA | Health | Examine whether care transition interventions that are congruent with the complexity of the processes and conditions they are trying to improve will have better outcomes. | 44 | Complex adaptive system theory | Scoring, Chi squared tests, Fisher’s exact test | - |
| Phillips and Merrill (2015) | USA | Health | Understand the potential barriers and challenges necessary to transform healthcare given its complex nonlinear behavior. | 477 | Complexity science | Directed content analysis | - |
| Pilgrim and Blum (2012a) | USA | Health | Identify risk and protective factors associated with adolescent mental and physical health in the English-speaking Caribbean. | 68 | Ecological systems theory | - | - |
| Pilgrim and Blum (2012b) | USA | Health | Identify risk and protective factors associated with adolescent sexual and reproductive health in the English-speaking Caribbean. | 30 | Ecological systems theory | - | - |
| Reid et al. (2017) | Australia | Health | Determine the optimal strategy and governance for the prevention and control of leptospirosis in Fiji. | - | Systems thinking | Realist synthesis | - |
| Riley et al. (2012) | Canada | Health | Identify useful principles to better understand, assess and shape the creation and use of evidence that will contribute to improved public health practice and policy. | 20 | Complexity science | Descriptive categorisation | - |
| Rogers et al. (2015) | USA | Health | Identify what causal influences on child language development (a) have been explicitly studied within key communication sciences and disorders (CSD) and (b) are being controlled for and discussed within CSD journal articles and to what extent is the possibility of gene–environment interplay being acknowledged. | 346 | Developmental systems theory | Categorization | - |
| Rogov and Rozenblat (2018) | Switzerland | Urban and rural studies | Understand the current state of research in urban resilience, its relations to urban sustainability and to integrate several distinct approaches into a multi-level perspective of cities comprising micro, meso and macro levels and their interactions. | 1012 | Complex systems approach | Conceptual maps | Urban and regional resilience research in a multi-level perspective |
| Sabri et al. (2013) | USA | Child and family studies | Examine research on children and adolescent’s exposure to violence in various settings (e.g., home, school, community) using Bronfenbrenner’s ecological systems framework. | 177 | Ecological systems theory | - | - |
| Sahin et al. (2013) | France | Health | Assess the potential factors that may generate complexity in managing operations in home care organizations. | - | Complexity | Content analysis | - |
| Salzmann-Erikson and Yifter (2019) | Sweden | Health | Identify and describe risk factors and triggers that may result in patient-initiated violence on inpatient psychiatric units. | 18 | Complexity science | Inductive data analysis | Complexity of risk factors and triggers |
| Shigayeva and Coker (2015) | UK | Health | Clarify the conceptual understanding of what constitutes sustainability and the link between notions of sustainability and integration. | 108 | Complex adaptive system theory | Thematic synthesis | Framework for analysis  of a programme’s sustainability |
| Simbizi et al. (2014) | Netherlands | Urban and rural studies | Revisit and refine the concept of tenure security in the context of Sub-Saharan Africa’s rural poor | 91 | System thinking | Critical descriptive analysis | Conceptual model of land tenure security |
| Snyder and Burke (1986) | USA | Education | Describe schools of allied health as organizational systems comprised of integrated components of task, structure, technology, and people. | 16 | Systems approach | - | - |
| Strom et al. (2018) | USA | Education | Build a more complex, nuanced, and layered understanding of the multidimensional influences that work together to shape the practices of novice teachers. | 46 | Complexity theory | Meta-synthesis | Systems of first-year teaching |
| Techera et al. (2016) | USA | Health | Create a systems model depicting causal factors of occupational fatigue, interrelationships among causal factors, outcomes of fatigue, and interrelationships among outcomes. | 23 | - | Meta-analysis | Systems model |
| Thoroman et al. (2018) | Australia | Organizational studies | Determine whether industry-level near miss reporting systems are consistent with systems thinking. | 20 | Rasmussen’s Risk Management Framework | Rating | - |
| Verhaeghe et al. (2005) | Belgium | Health | Structure the available information on the psychological reactions of family members confronted with traumatic brain injury. | 94 | Systems theory | - | - |
| Voisin et al. (2012) | USA | Child and family studies | Identify correlates of risky and STIs among detained youth. | 48 | Ecological systems theory | - | - |
| Weng et al. (2016) | China | Child and family studies | Explore the factors related to Chinese juvenile delinquent behaviors. | 45 | Ecological systems theory | Tabulation and classification | - |
| Werner and Holden (2015) | USA | Health | Develop a sociotechnical systems model of interruption in the emergency department. | 15 | Sociotechnical systems models | - | Socio-technical system model of interrupted task performance in the wild |

**Supplementary file 2.** Characteristics of Included Papers using systems methods (n=22)

| **Authors**  **(date)** | | **Country of lead author** | **Field** | **Aim** | **Number of papers included** | **Methods and methodologies** | **Systems visuali-zation** | **Software used** | **Other data than literature review** |
| --- | --- | --- | --- | --- | --- | --- | --- | --- | --- |
| Abotaleb and El-adaway (2018) | | USA | Organizational studies | Grasp in a holistic manner the direct and indirect impacts of different construction project parameters. | 55 studies (QT) | Social network analysis  Simplified analysis | CLD | - | - |
| Adamu et al. (2019) | | South Africa | Health | Estimate the prevalence of missed opportunities for vaccination (MOV) among children in Africa and explore the factors responsible for MOV. | 20 studies (QT) | SD  Meta-analysis | CLD | Vensim | - |
| Azevedo and Saurin (2018) | | Brazil | Environmental studies | Identify the factors that influence the complexity of water distributin systems (WDSs), and understand how the methods used to control losses in WDSs account for complexity. | 49 studies (QT) | SD  Content analysis | CLD | - | - |
| Brous et al. (2019) | | Netherlands | Information sciences | Develop an extensible model of asset management data infrastructures. | 18 papers | Matrix of concepts (grouped key concepts)  Cross-over modeling technique | data model | - | case study |
| Brzezina et al. (2016) | | Belgium | Environmental studies | Identify the vulnerabilities of the conventional food system that result from both its internal structure and exposure to external disturbances.  Evaluate whether organic farming can reduce  the vulnerabilities. | N/A | SD | CLD | Vensim | time series data |
| Chidumayo (2018) | | Zambia | Health | Explore the effect of dog demography on rabies vaccination coverage following a vaccination campaign with ≥70% vaccination coverage. | 22 studies (QT) | SD | SFD | Vensim | district data |
| Dicks et al. (2017) | | Australia | Health | Gain a holistic understanding of family experiences during of a potential organ donation. | 7 SR | System of systems framework  Grounded theory | Systemic map | Kumu systems mapping tool | - |
| Eickhoff et al. (2009) | | Germany | Health | Build a system model of effective connectivity in the human brain underlying overt speech production. | 19 studies (QT) | SD  Activation likelihood estimation meta-analysis | System model | - | imaging data using Bayesian model selection |
| Frerichs (2015); Frerichs et al. (2015) (dissertation) | | USA | Health | Elucidate the contribution of school design to a systems approach to obesity prevention. | 102 studies (QL, QL & MM) | SD | CLD | - | mixed methods studies |
| Garcia et al. (2017) | | Brazil | Physical activity | Create a model depicting the interactions between key elements of leisure-time physical activity in adults. | 19 SR | ABM | Dynamic conceptual model | - | experts consultations |
| Guo et al. (2014) | | UK | Health | Create a model on the key factors which may cause diagnostic errors, and their interrelationships. | 57 studies (QT) | SD | CLD | - | clinicians consultations |
| Hagg (2013); Woodward-Hagg and Bar-On (2014) (dissertation) | | USA | Organizational studies | Provide an understanding of the mechanisms that drive successful, sustained, enterprise-level transformation. | 49 papers | SD  Realist synthesis (thematic synthesis,  cross-case comparison) | CLD  SFD | - | case studies |
| Jalali (2015) (dissertation) | | USA | Health | Understand the dynamics of obesity interventions. | 21 studies (QT) | SD | CLD | - | case studies |
| Kumar and Thakkar (2017) | | India | Organizational studies | Explore the dynamic relationships involved within the technological and economic factors of R&D project. | N/A | SD  Analytic network process | SFD | Vensim | experts consultations,  case study |
| Martinez Garcia and Sheehan (2016) | | Spain | Health | Identify vulnerabilities, risk factors and capacities or resilience factors of children in the face of extreme weather events driven disasters. | N/A  studies and reviews | SD | CLD | Vensim | - |
| Oesterreich and Teuteberg (2018) | | Germany | Organizational studies | Develop a quantification model for the financial assessment of the specific augmented reality application scenario. | N/A  (QT) | SD | CLD  SFD | Vensim | artificial data |
| Rushton and Lindsay (2003) | | UK | Education | Explore the complex area of clinical education through a multilevel analysis. | N/A | Soft system methodology  Content analysis | Open system representa-tion | - | - |
| Schuh et al. (2017) | | USA | Health | Demonstrate how public health and systems science methods can be combined to examine the structure and behavior of Afghanistan’s routine childhood immunization system. | N/A | SD | CLD  SFD | Vensim | survey data, expert consultations |
| Sylvester et al. (2013) | | New Zealand | Information sciences | Examine the nature, role and function of the literature review in academic discourse. | 87 | Soft system analysis (CATWOE)  Meta-narrative synthesis | Rich picture | - | - |
| Xie et al. (2017) | | China | Health | Better understand the One Health concept by providing a systematic representation of the components and their interactions. | 19 studies and SR | SD | CLD | AnyLogic PLE | - |
| Yawson (2017); Yawson and Greiman (2016) | | USA | Environmental studies | Determine the current and future skill needs of the emerging agrifood nanotechnology sector. | 53 studies (QL, QT), policy analysis and reviews | SD (System mapping)  Qualitative systems analysis  Descriptive statistics Strategic flexibility analysis | CLD | Vensim | surveys and interviews |
| Zammar et al. (2010) | | Brazil | Health | Understand the factors affecting the willingness to participate in clinical trials in Brazil and India. | 5 studies (QT) | SD | SFD | Vensim | - |
| Acronyms and abbreviations: ABM: agent-based modeling; CLD: causal loop diagram; MM: mixed methods; N/A: non-available; QL: qualitative; QT: quantitative; SFD: stock-and-flow diagram; SD: systems dynamics; SR: systematic reviews. | | | | | | | | | |
|  |  | | | | | | | | |

**References**

Abotaleb, I. S., El-adaway, I. H. (2018). Managing construction projects through dynamic modeling: Reviewing the existing body of knowledge and deriving future research directions. *Journal of Management in Engineering, 34*(6), 04018033.

Adamu, A. A., Sarki, A. M., Uthman, O. A., Wiyeh, A. B., Gadanya, M. A., Wiysonge, C. S. (2019). Prevalence and dynamics of missed opportunities for vaccination among children in Africa: Applying systems thinking in a systematic review and meta-analysis of observational studies. *Expert Review of Vaccines, 18*(5), 547-558.

Aksulu, A., Wade, M. (2010). A comprehensive review and synthesis of open source research. *Journal of the Association for Information Systems, 11*(11), 576-656.

Alexander, A., Walker, H., Naim, M. (2014). Decision theory in sustainable supply chain management: A literature review. *Supply Chain Management-an International Journal, 19*(5-6), 504-522.

Azevedo, B. B., Saurin, T. A. (2018). Losses in water distribution systems: A complexity theory perspective. *Water Resources Management, 32*(9), 2919-2936.

Basurto, X., Gelcich, S., Ostrom, E. (2013). The social ecological system framework as a knowledge classificatory system for benthic small-scale fisheries. *Global Environmental Change-Human and Policy Dimensions, 23*(6), 1366-1380.

Benton, D. C., Gonzalez-Jurado, M. A., Beneit-Montesinos, J. V., Fernandez Fernandez, P. (2013). Use of open systems theory to describe regulatory trends. *Journal of Nursing Regulation, 4*(3), 49-56.

Best, A., Greenhalgh, T., Lewis, S., Saul, J. E., Carroll, S., Bitz, J. (2012). Large-system transformation in health care: A realist review. *Milbank Quarterly, 90*(3), 421-456.

Bowen, D. J., Barrington, W. E., Beresford, S. A. A. (2015). Identifying the effects of environmental and policy change interventions on healthy eating. *Annual Review of Public Health, 36*, 289-306.

Brous, P., Janssen, M., Herder, P. (2019). Next generation data infrastructures: Towards an extendable model of the asset management data infrastructure as complex adaptive system. *Complexity, 2019*, 1-17.

Brzezina, N., Kopainsky, B., Mathijs, E. (2016). Can organic farming reduce vulnerabilities and enhance the resilience of the european food system? A critical assessment using system dynamics structural thinking tools. *Sustainability, 8*(10), 32.

Burns, M. K., Warmbold-Brann, K., Zaslofsky, A. F. (2015). Ecological systems theory in school psychology review. *School Psychology Review, 44*(3), 249-261.

Butt, G., Markle-Reid, M., Browne, G. (2008). Interprofessional partnerships in chronic illness care: A conceptual model for measuring partnership effectiveness. *International Journal of Integrated Care, 8*, 1-14.

Cameron, A. A., Calderwood, K., McMurphy, S. (2018). A systematic literature review of the etiology of borderline personality disorder from an ecological systems perspective. *Social Work in Mental Health, 17*(3), 364-380.

Cassarino, M., Murphy, G. (2018). Reducing young drivers' crash risk: Are we there yet? An ecological systems-based review of the last decade of research. *Transportation Research Part F: Traffic Psychology and Behaviour, 56*, 54-73.

Chidumayo, N. N. (2018). System dynamics modelling approach to explore the effect of dog demography on rabies vaccination coverage in Africa. *PloS One, 13*(10), e0205884.

Dallat, C., Salmon, P. M., Goode, N. (2017). Risky systems versus risky people: To what extent do risk assessment methods consider the systems approach to accident causation? A review of the literature. *Safety Science, 119*, 266-279.

Davidson, P. L. (1997). *A theoretical values-based model for parametric organizational destochastization.* (doctoral dissertation), Fielding Institute, Santa Barbara, CA.

Dicks, S. G., Ranse, K., van Haren, F. M., Boer, D. P. (2017). In-hospital experiences of families of potential organ donors: A systematic review and qualitative synthesis. *Health Psychology Open, 4*(2), 2055102917709375.

Dresch, A., Collatto, D. C., Lacerda, D. P. (2018). Theoretical understanding between competitiveness and productivity: Firm level. *Ingeniería y Competitividad, 20*(2), 69-86.

Duckworth, A. L., Azulay Cbertok, l. R. (2012). Review of perinatal partner-focused smoking cessation interventions. *MCN: The American Journal of Maternal Child Nursing, 37*(3), 174-181.

Eickhoff, S. B., Heim, S., Zilles, K., Amunts, K. (2009). A systems perspective on the effective connectivity of overt speech production. *Philosophical Transactions of the Royal Society London, 367*(1896), 2399-2421.

Eklund, R., Salzmann-Erikson, M. (2016). An integrative review of the literature on how eating disorders among adolescents affect the family as a system - Complex structures and relational processes. *Mental Health Review Journal, 21*(3), 213-230.

El-Behadli, A. F., Sharp, C., Hughes, S. O., Obasi, E. M., Nicklas, T. A. (2015). Maternal depression, stress and feeding styles: Towards a framework for theory and research in child obesity. *British Journal of Nutrition, 113 Suppl*, S55-71.

Elnitsky, C. A., Blevins, C. L., Fisher, M. P., Magruder, K. (2017). Military service member and veteran reintegration: A critical review and adapted ecological model. *American Journal of Orthopsychiatry, 87*(2), 114-128.

Frerichs, L. (2015). *Architecture and design for healthy eating in schools.* (doctoral dissertation), University of Nebraska, Omaha, NE.

Frerichs, L., Brittin, J., Sorensen, D., Trowbridge, M. J., Yaroch, A. L., Siahpush, M., et al. (2015). Influence of school architecture and design on healthy eating: A review of the evidence. *American Journal of Public Health, 105*(4), e46-57.

Fulgencio, H. T., Orij, R., Le Fever, H. (2016). Mapping and conceptualizing the measurement of organizational social value using systems thinking. *European Public & Social Innovation Review, 1*(1), 17-31.

Furst, M. A., Gandre, C., Romero Lopez-Alberca, C., Salvador-Carulla, L. (2019). Healthcare ecosystems research in mental health: A scoping review of methods to describe the context of local care delivery. *BMC Health Services Research, 19*(1), 173.

Garcia, L. M. T., Roux, A. V. D., Martins, A. C. R., Yang, Y., Florindo, A. A. (2017). Development of a dynamic framework to explain population patterns of leisure-time physical activity through agent-based modeling. *International Journal of Behavioral Nutrition and Physical Activity, 14*, 111.

Gissi, E., Fraschetti, S., Micheli, F. (2019). Incorporating change in marine spatial planning: A review. *Environmental Science and Policy, 92*, 191-200.

Goode, N., Newnam, S., Salmon, P. M. (2019). Musculoskeletal disorders in the workplace: Development of a systems thinking-based prototype classification scheme to better understand the risks. *Safety Science, 120*, 146-156.

Greenlee, J. L., Winter, M. A., Diehl, J. J. (2018). Family level processes associated with outcomes for individuals with autism spectrum disorder: A scoping review. *Research in Autism Spectrum Disorders, 53*, 41-52.

Guo, S., Roudsari, A., Garcez, A. (2014). A causal loop approach to the study of diagnostic errors. *Studies in Health Technology and Informatics, 205*, 73-77.

Hagg, H. (2013). *Large system transformation within healthcare organizations utilizing Lean deployment strategies.* (doctoral dissertation), Worcester Polytechnic Institute, Worcester, MA.

Hamidi, H. (2018). Research possibilities in the field of human resource development in small - medium enterprises in developing countries: A review and research agenda. *Global Business and Management Research, 10*(2), 146-156.

Hirano, K. A., Rowe, D., Lindstrom, L., Chan, P. (2018). Systemic barriers to family involvement in transition planning for youth with disabilities: A qualitative metasynthesis. *Journal of Child and Family Studies, 27*, 3440–3456.

Holdsworth, M., Nicolaou, M., Langoien, L. J., Osei-Kwasi, H. A., Chastin, S. F. M., Stok, F. M., et al. (2017). Developing a systems-based framework of the factors influencing dietary and physical activity behaviours in ethnic minority populations living in Europe - a DEDIPAC study. *International Journal of Behavioral Nutrition & Physical Activity, 14*(1), 154.

Holloway Cripps, K. G. (2016). *Too hot, too cold, and then just right: Balancing form and function in biophilic designed office workspaces.* (doctoral dissertation), University of Maryland University College, Maryland.

Hong, J. S., Huang, H., Sabri, B., Kim, J. S. (2011). Substance abuse among Asian American youth: An ecological review of the literature. *Children and Youth Services Review, 33*(5), 669-677.

Hong, J. S., Kral, M. J., Espelage, D. L., Allen-Meares, P. (2012). The social ecology of adolescent-initiated parent abuse: A review of the literature. *Child Psychiatry & Human Development, 43*(3), 431-454.

Jackson, V., Chou, S., Browne, K. (2017). Protective factors against child victimization in the school and community: An exploratory systematic review of longitudinal predictors and interacting variables. *Trauma Violence & Abuse, 18*(3), 303-321.

Jalali, S. M. J. (2015). *Three essays on systems thinking and dynamic modeling in obesity prevention interventions.* (doctoral dissertation), Virginia Polytechnic Institute and State University, Falls Church, VA.

Javadi, D., Feldhaus, I., Mancuso, A., Ghaffar, A. (2017). Applying systems thinking to task shifting for mental health using lay providers: A review of the evidence. *Global Mental Health, 4*, e14.

Kennedy, H., DeChants, J., Bender, K., Anyon, Y. (2019). More than data collectors: A systematic review of the environmental outcomes of youth inquiry approaches in the United States. *American Journal of Community Psychology, 63*(1-2), 208-226.

Knocke, J., Schuster, T. (2017). Repatriation of international assignees. *Journal of Global Mobility, 5*(3), 275-303.

Kuch, B. (2017). The relations between ecological sustainability and geographical proximity: A review of the literature. *The International Journal of Technology Management & Sustainable Development, 16*(1), 97-114.

Kuhnen, M., Hahn, R. (2018). Systemic social performance measurement: Systematic literature review and explanations on the academic status quo from a product life-cycle perspective. *Journal of Cleaner Production, 205*, 690-705.

Kumar, S., Thakkar, J. J. (2017). Schedule and cost overrun analysis for R&D projects using ANP and system dynamics. *International Journal of Quality & Reliability Management, 34*(9), 1551-1567.

Kupers, E., Lehmann-Wermser, A., McPherson, G., van Geert, P. (2019). Children's creativity: A theoretical framework and systematic review. *Review of Educational Research, 89*(1), 93-124.

Kusuwo, P., Myezwa, H., Pilusa, S., M'Kumbuzi, V. (2017). A systematic review to identify system-related elements that can be used to evaluate community-based rehabilitation (CBR) programmes. *European Journal of Physiotherapy, 19 (Supplement 1)*, 41-46.

Lapalme, J., Bisset, S., Potvin, L. (2014). Role of context in evaluating neighbourhood interventions promoting positive youth development: A narrative systematic review. *International Journal of Public Health, 59*(1), 31-42.

Leykum, L. K., Parchman, M., Pugh, J., Lawrence, V., Noel, P. H., McDaniel Jr, R. R. (2010). The importance of organizational characteristics for improving outcomes in patients with chronic disease: A systematic review of congestive heart failure. *Implementation Science, 5*, 66.

Leykum, L. K., Pugh, J., Lawrence, V., Parchman, M., Noel, P. H., Cornell, J., et al. (2007). Organizational interventions employing principles of complexity science have improved outcomes for patients with Type II diabetes. *Implementation Science, 2*, 28.

Liao, M., Lee, A. S., Roberts-Lewis, A. C., Hong, J. S., Jiao, K. (2011). Child maltreatment in China: An ecological review of the literature. *Children and Youth Services Review, 33*(9), 1709-1719.

Lorthios-Guilledroit, A., Richard, L., Filiatrault, J. (2018). Factors associated with the implementation of community-based peer-led health promotion programs: A scoping review. *Evaluation & Program Planning, 68*, 19-33.

MacDonald, J.-A., Davies, B., Edwards, N., Marck, P., Guernsey, J. R. (2012a). Priority setting and policy advocacy for community environmental health by nursing associations: A conceptual framework to guide research. *Aporia, 4*(1), 18-19.

MacDonald, J. A., Edwards, N., Davies, B., Marck, P., Guernsey, J. R. (2012b). Priority setting and policy advocacy by nursing associations: A scoping review and implications using a socio-ecological whole systems lens. *Health Policy, 107*(1), 31-43.

Marek, M. W., Wu, W. C. V. (2014). Environmental factors affecting computer assisted language learning success: A Complex Dynamic Systems conceptual model. *Computer Assisted Language Learning, 27*(6), 560-578.

Marheineke, M., Velamuri, V. K., Moslein, K. M. (2016). On the importance of boundary objects for virtual collaboration: A review of the literature. *Technology Analysis & Strategic Management, 28*(9), 1108-1122.

Martinez Garcia, D., Sheehan, M. C. (2016). Extreme weather-driven disasters and children’s health. *International Journal of Health Services, 46*(1), 79-105.

McLain, R., Lawry, S., Ojanen, M. (2018). Fisheries' property regimes and environmental outcomes: A realist synthesis review. *World Development, 102*, 213-227.

Meade, C. S., Ickovics, J. R. (2005). Systematic review of sexual risk among pregnant and mothering teens in the USA: Pregnancy as an opportunity for integrated prevention of STD and repeat pregnancy. *Social Science & Medicine, 60*(4), 661-678.

Merali, Y., Papadopoulos, T., Nadkarni, T. (2012). Information systems strategy: Past, present, future? *Journal of Strategic Information Systems, 21*(2), 125-153.

Miles, E. M., Narayan, A. J., Watamura, S. E. (2019). Syrian caregivers in perimigration: A systematic review from an ecological systems perspective. *Translational Issues in Psychological Science, 5*(1), 78-90.

Minh, A., Muhajarine, N., Janus, M., Brownell, M., Guhn, M. (2017). A review of neighborhood effects and early child development: How, where, and for whom, do neighborhoods matter? *Health & Place, 46*, 155-174.

Mitchell, M. L., Courtney, M., Coyer, F. (2003). Understanding uncertainty and minimizing families' anxiety at the time of transfer from intensive care. *Nursing & Health Sciences, 5*(3), 207-217.

Newig, J., Fritsch, O. (2009). Environmental governance: Participatory, multi-level - and effective? *Environmental Policy and Governance, 19*(3), 197.

Noyes, J., Brenner, M., Fox, P., Guerin, A. (2014). Reconceptualizing children's complex discharge with health systems theory: Novel integrative review with embedded expert consultation and theory development. *Journal of Advanced Nursing, 70*(5), 975-996.

O'Brien-Pallas, L., Meyer, R. M., Hayes, L. J., Wang, S. (2011). The Patient Care Delivery Model - an open system framework: Conceptualisation, literature review and analytical strategy. *Journal of Clinical Nursing, 20*(11-12), 1640-1650.

Odom, S. L., Vitztum, J., Wolery, R., Lieber, J., Sandall, S., Hanson, M. J., et al. (2004). Preschool inclusion in the United States: A review of research from an ecological systems perspective. *Journal of Research in Special Educational Needs, 4*(1), 17-49.

Oesterreich, T. D., Teuteberg, F. (2018). Why one big picture is worth a thousand numbers: Measuring intangible benefits of investments in augmented reality based assistive technology using utility effect chains and system dynamics. *Information Systems and E-Business Management, 16*(2), 407-441.

Okwir, S., Nudurupati, S. S., Ginieis, M., Angelis, J. (2018). Performance measurement and management systems: A perspective from complexity theory. *International Journal of Management Reviews, 20*(3), 731-754.

Penney, L. S., Nahid, M., Leykum, L. K., Lanham, H. J., Noel, P. H., Finley, E. P., et al. (2018). Interventions to reduce readmissions: Can complex adaptive system theory explain the heterogeneity in effectiveness? A systematic review. *BMC Health Services Research, 18*(1), 894.

Phillips, A. B., Merrill, J. A. (2015). Innovative use of the integrative review to evaluate evidence of technology transformation in healthcare. *Journal of Biomedical Informatics, 58*, 114-121.

Pilgrim, N. A., Blum, R. W. (2012a). Adolescent mental and physical health in the English-speaking Caribbean. *Revista Panamericana de Salud Publica, 32*(1), 62-91.

Pilgrim, N. A., Blum, R. W. (2012b). Protective and risk factors associated with adolescent sexual and reproductive health in the English-speaking Caribbean: A literature review. *Journal of Adolescent Health, 50*(1), 5-23.

Reid, S. A., Rodney, A., Kama, M., Hill, P. S. (2017). A process for developing multisectoral strategies for zoonoses: The case of leptospirosis in Fiji. *BMC Public Health, 17*(1), 671.

Riley, B., Norman, C. D., Best, A. (2012). Knowledge integration in public health: A rapid review using systems thinking. *Evidence & Policy, 8*(4), 417-431.

Rogers, C. R., Nulty, K. L., Betancourt, M. A., DeThorne, L. S. (2015). Causal effects on child language development: A review of studies in communication sciences and disorders. *Journal of Communication Disorders, 57*, 3-15.

Rogov, M., Rozenblat, C. (2018). Urban resilience discourse analysis: Towards a multi-level approach to cities. *Sustainability, 10*(12), 21.

Rushton, A., Lindsay, G. (2003). Clinical education: A critical analysis using soft systems methodology. *International Journal of Therapy & Rehabilitation, 10*(6), 271-280.

Sabri, B., Hong, J. S., Campbell, J. C., Cho, H. (2013). Understanding children and adolescents' victimizations at multiple levels: An ecological review of the literature. *Journal of Social Service Research, 39*(3), 322-334.

Sahin, E., Vidal, L. A., Benzarti, E. (2013). A framework to evaluate the complexity of home care services. *Kybernetes, 42*(4), 569-592.

Salzmann-Erikson, M., Yifter, L. (2019). Risk factors and triggers that may result in patient-initiated violence on inpatient psychiatric units: An integrative review. *Clinical Nursing Research*, 1054773818823333.

Schuh, H. B., Merritt, M. W., Igusa, T., Lee, B. Y., Peters, D. H. (2017). Examining the structure and behavior of Afghanistan's routine childhood immunization system using system dynamics modeling. *International Journal of Health Governance, 22*(3), 212-227.

Shigayeva, A., Coker, R. J. (2015). Communicable disease control programmes and health systems: An analytical approach to sustainability. *Health Policy & Planning, 30*(3), 368-385.

Simbizi, M. C. D., Bennett, R. M., Zevenbergen, J. (2014). Land tenure security: Revisiting and refining the concept for Sub-Saharan Africa's rural poor. *Land Use Policy, 36*, 231-238.

Snyder, J. R., Burke, J. E. (1986). Systems analysis of schools of allied health using Leavitt's Organizational Model: A meta-analysis. *Journal of Allied Health, 15*(2), 153-161.

Strom, K. J., Martin, A. D., Villegas, A. M. (2018). Clinging to the edge of chaos: The emergence of practice in the first year of teaching. *Teachers College Record, 120*(7), 1-32.

Sylvester, A., Tate, M., Johnstone, D. (2013). Beyond synthesis: Re-presenting heterogeneous research literature. *Behaviour & Information Technology, 32*(12), 1199-1215.

Techera, U., Hallowell, M., Stambaugh, N., Littlejohn, R. (2016). Causes and consequences of occupational fatigue: Meta-analysis and systems model. *Journal of Occupational and Environmental Medicine, 58*(10), 961-973.

Thoroman, B., Goode, N., Salmon, P. (2018). System thinking applied to near misses: A review of industry-wide near miss reporting systems. *Theoretical Issues in Ergonomics Science, 19*(6), 712-737.

Verhaeghe, S., Defloor, T., Grypdonck, M. (2005). Stress and coping among families of patients with traumatic brain injury: A review of the literature. *Journal of Clinical Nursing, 14*(8), 1004-1012.

Voisin, D. R., Hong, J. S., King, K. (2012). Ecological factors associated with sexual risk behaviors among detained adolescents: A systematic review. *Children & Youth Services Review, 34*(10), 1983-1991.

Weng, X., Ran, M. S., Chui, W. H. (2016). Juvenile delinquency in Chinese adolescents: An ecological review of the literature. *Aggression and Violent Behavior, 31*, 26-36.

Werner, N. E., Holden, R. J. (2015). Interruptions in the wild: Development of a sociotechnical systems model of interruptions in the emergency department through a systematic review. *Applied Ergonomics, 51*, 244-254.

Woodward-Hagg, H., Bar-On, I. (2014). Large system transformation within healthcare organizations utilizing lean deployment strategies. *IIE Annual Conference. Proceedings*, 458-467.

Xie, T., Liu, W., Anderson, B. D., Liu, X., Gray, G. C. (2017). A system dynamics approach to understanding the One Health concept. *PloS One, 12*(9), e0184430.

Yawson, R. M. (2017). Systematic review to identify skill needs for agrifood nanotechnology workforce. *Career and Technical Education Research, 42*(3), 149-181.

Yawson, R. M., Greiman, B. C. (2016). A systems approach to identify skill needs for agrifood nanotechnology: A multiphase mixed methods study. *Human Resource Development Quarterly, 27*(4), 517-545.

Zammar, G., Meister, H., Shah, J., Phadtare, A., Cofiel, L., Pietrobon, R. (2010). So different, yet so similar: Meta-analysis and policy modeling of willingness to participate in clinical trials among Brazilians and Indians. *PloS One, 5*(12), e14368.
